# Differential Proteome Analysis of Hybrid Bamboo (*Bambusa pervariabilis × Dendrocalamopsis grandis*) Under Fungal Stress (*Arthrinium phaeospermum*)

**DOI:** 10.1038/s41598-019-55229-0

**Published:** 2019-12-10

**Authors:** Shujiang Li, Xinmei Fang, Shan Han, Tianhui Zhu, Hanmingyue Zhu

**Affiliations:** College of Forestry, Sichuan Agricultural University, No. 211, Huimin Road, Wenjiang District, Chengdu, 611130 Sichuan Province China

**Keywords:** Computational biology and bioinformatics, Plant sciences

## Abstract

In this study, TMT (tandem mass tag)-labeled quantitative protein technology combined with LC–MS/MS (liquid chromatography-mass spectrometry/mass spectrometry) was used to isolate and identify the proteins of the hybrid bamboo (*Bambusa pervariabilis* × *Dendrocalamopsis grandis*) and the bamboo inoculated with the pathogenic fungi *Arthrinium phaeospermum*. A total of 3320 unique peptide fragments were identified after inoculation with either *A. phaeospermum* or sterile water, and 1791 proteins were quantified. A total of 102 differentially expressed proteins were obtained, of which 66 differential proteins were upregulated and 36 downregulated in the treatment group. Annotation and enrichment analysis of these peptides and proteins using the GO (Gene Ontology) and KEGG (Kyoto Encyclopedia of Genes and Genomes) databases with bioinformatics software showed that the differentially expressed protein functional annotation items were mainly concentrated on biological processes and cell components. The LC–PRM/MS (liquid chromatography-parallel reaction monitoring/mass spectrometry) quantitative analysis technique was used to quantitatively analyze 11 differential candidate proteins obtained by TMT combined with LC–MS/MS. The up–down trend of 10 differential proteins in the PRM results was consistent with that of the TMT quantitative analysis. The coincidence rate of the two results was 91%, which confirmed the reliability of the proteomic results. Therefore, the differentially expressed proteins and signaling pathways discovered here may be the further concern for the bamboo-pathogen interaction studies.

## Introduction

The term “proteome” was first proposed in 1995, originating from the hybridization of “protein” with “genome”, and refers to “all proteins expressed in a cell or an organism”^1,2^. The characterization of proteomes has since contributed substantially to our understanding of different diseases, with the aim of gaining insight into the cellular signaling pathways underlying disease and the discovery of novel biomarkers for screening, early detection, and diagnosis, as well as to determine and predict the responses to specific treatments^3^. Compared to the static nature of the genome, the plant proteome is highly complex and dynamic. As proteins are an important part of the main signal transduction and biochemical pathways, studying the protein abundance is essential for revealing the molecular mechanism of plant growth, development, and interaction with the environment^4^. Quantitative proteomics refers to the mass spectrometry detection of specific known proteins without the need for the full detection of all unknown proteins.

Two kinds of innate immune mechanisms have formed during the long-term co-evolution between plants and pathogens. Pathogen-associated molecular pattern (PAMP)-induced resistance mechanisms (PAMP-triggered immunity, PTI), and the pathogen effector protein (Effector)-induced resistance mechanisms (effector-triggered immunity, ETI). PTI recognizes PAMPs-a conservative structural molecule of pathogens-by pattern recognition receptors (PRRs) on the plant cell surface, which then activates basic defense responses in plants. Chitin and dextran from pathogenic fungi are examples of PAMPs that induce PTI responses in plants^5^. In *Arabidopsis thaliana*, chitin can be recognized, bound, and activated by receptor-like protein kinase CERK1 (chitin elicitor receptor kinase 1) containing the LysM domain^6^. Studies have shown that OsCEBiP (hychitin elicitor binding protein) and LysM domain proteins LYP4 and LYP6 (lysin motif-containing proteins) are involved in the identification of chitin in the cell wall of pathogenic fungi^7^. ETI relies on plant disease resistance proteins (R proteins) recognizing the effector proteins secreted by pathogens either directly or indirectly, thus triggering a strong resistance response to inhibit the infection of pathogens, which usually manifests as a HR (hypersensitive response). In rice, AvrPiz-t, a nontoxic effector protein of *Magnaporthe grisea*, significantly inhibited the PTI response induced by flagellin and chitin. It was found that AvrPiz-t inhibited the PTI response mediated by APIP6 by interfering with the activity of ubiquitin ligase APIP6^8^, revealing that effector proteins of pathogenic bacteria could inhibit plant disease resistance by interfering with the degradation system of ubiquitin proteins.

Technological developments make MS (mass spectrometry)-based proteomics a central pillar of biochemical research. At present, iTRAQ (isobaric tags for relative and absolute quantitation) technology and TMT (tandem mass tag) technology are the main technologies used for the quantitative analysis of differential proteomics mass spectrometry^9,10^. Particularly, TMT chemical labeling quantitative proteomics technology has become a very important mass spectrometry quantitative method^11^. In order to reveal *Brassica napus* defense mechanisms against *Sclerotinia sclerotiorum*, the proteomes of *B. napus* leaves inoculated with *S. sclerotiorum* wild-type strain 1980 and nonpathogenic mutant strain Ep-1PB, as well as an empty agar plug as the control, were analyzed using the TMT label-based quantitative analysis technique. In total, there were 79 differentially expressed proteins in the nonpathogenic mutant strain EP-1pb and empty agar plug, 299 differentially expressed proteins in the *S. sclerotiorum* wild-type strain 1980 and empty agar plug, and 173 differentially expressed proteins in the *S. sclerotiorum* wild-type strain 1980 and nonpathogenic mutant strain EP-1pb. The differential expression of 12 selected proteins was confirmed by RT-qPCR (real-time fluorescence quantitative) analysis. This provides a new molecular mechanism for the defense response of *Brassica napus* to *S. sclerotiorum* and helps to screen for resistant proteins^12^. Liu *et al*. used TMT quantitative proteomics coupled with UPLC MS/MS (ultra-performance liquid chromatography mass spectrometry/mass spectrometry) to analyze the physiology and proteomics of tea plants treated with different fluorides, and analyzed the molecular mechanism of fluoride accumulation/detoxification in tea plants^13^. PRM (parallel reaction monitoring) technology is an important method in the current targeted proteomics research, allowing simultaneous monitoring of all transitions as a full MS/MS scanning profile and thus provides an enhanced selectivity and confidence in quantitation of each analyzed target protein^14^. Recently, this technology combined with iTRAQ or TMT is widely used in the proteome in the pharmaceutical and environmental industries^15–17^. However, there is few studies in the area of plant, especially in plant-pathogen interactions^18,19^.

*Bambusa pervariabilis* McClure × *Dendrocalamopsis grandis* (Q.H.Dai & X.l.Tao ex Keng f.) Ohrnb., which is a species of hybrid bamboo with *B. pervaiabilis* and *D. grandis* as the male and female parents, has been extensively planted in southern China due to its characteristically advantageous abilities of growth and reproduction. It is often involved in the process of afforestation along rivers, which not only increases overall bamboo resources, but also conserves water and soil and improves the ecology in multiple different environments^20^, including the reinstatement of farmland and ecological forest construction along the Changjiang river basin in China^21^. However, one destructive disease, hybrid bamboo blight occurs in many provinces of China, causing the dead area of hybrid bamboo to reach 3000 hm2. Zhu *et al*.^22,23^, members of our research term, affirmed that *Arthrinium phaeospermum* (Corda) Elli started to infect the bamboo through conidia from April to May, spreading between individuals. The disease outbreak occurred in August-September, overwintered in October, and proceeded to infect more bamboo via conidia in the wind and rain in the second year. The pathogen belongs to Fungi, Dikarya, Ascomycota, Pezizomycotina, Sordariomycetes, Xylariomycetidae, Xylariales, Apiosporaceae, *Arthrinium*^24^. In our previous studies, the pathogenic toxin of *A. phaeospermum* had been clarified^21,25,26^, and the metabolomics responses of the bamboo to pathogenic fungal stress has been achieved by us this year^27^. However, the substrate of the metabolic pathways and the protein-substances of the metabolites remain unknown.

Pathogen-related molecular patterns and effector proteins can be recognized by plant surface pattern receptors and disease-resistant proteins, which can stimulate the resistance reaction of corresponding resistance proteins in plants to inhibit the infection of pathogens. Therefore, it is of great significance to search for disease resistance protein genes of bamboo shoot blight by comparing the proteomes of hybrid bamboo when inoculated with either *A. phaeospermum* or sterile water. In this study, the differential expression of proteins in hybrid bamboo inoculated with either pathogenic fungus *A. phaeospermum* or sterile water as a control were studied by using TMT protein quantitative technology and LC–MS/MS mass spectrometry. Then, PRM technology was used to quantitatively characterize target proteins with important biological functions among the differentially expressed proteins. We aimed to dissect the network of protein changes associated with the plant-pathogen interaction, thus deepening our understanding of its mechanism at the molecular level, and providing insights and guidance to control hybrid bamboo blight.

## Results

### Quantitative results of TMT

Protein enzymatic hydrolysis, peptide marker classification, and mass spectrometry were performed using TMT to identify and quantify protein segments and to analyze differentially expressed proteins. A total of 3320 unique peptide fragments were identified after inoculation with *A. phaeospermum* or sterile water, and 1791 proteins were quantified (Table 1). Therefore, TMT-labeling combined with mass spectrometry could effectively isolate and identify proteins from the hybrid bamboo inoculated with either *A. phaeospermum* or sterile water. When considering proteins whose abundance significantly differed by more than 1.2 times (up–down) (P value < 0.05), 102 differentially expressed proteins were identified in hybrid bamboo inoculated with *A. phaeospermum* in comparison with sterile water, of which 66 protein were upregulated while 36 were downregulated. The up-regulation and down-regulation parameters of differential proteins were shown in Table S1. The results showed that the most up-regulated proteins belonged Thaumatin family, with the ID PH01000846G0450 and the fold-change value was 5.480. Moreover, the most down-regulated protein was Chlorophyll A-B binding protein, with the ID PH01000947G0680 and the fold-change value was 0.398.

**Table 1:** Protein identification results statistics.

Identification result	Unique Peptide	Quantified protein	Up-regulated	Down -regulated	Significant difference in total protein
Total	3320	1791	66	36	102

### GO and KEGG analysis of differentially expressed proteins

**Figure 1:**
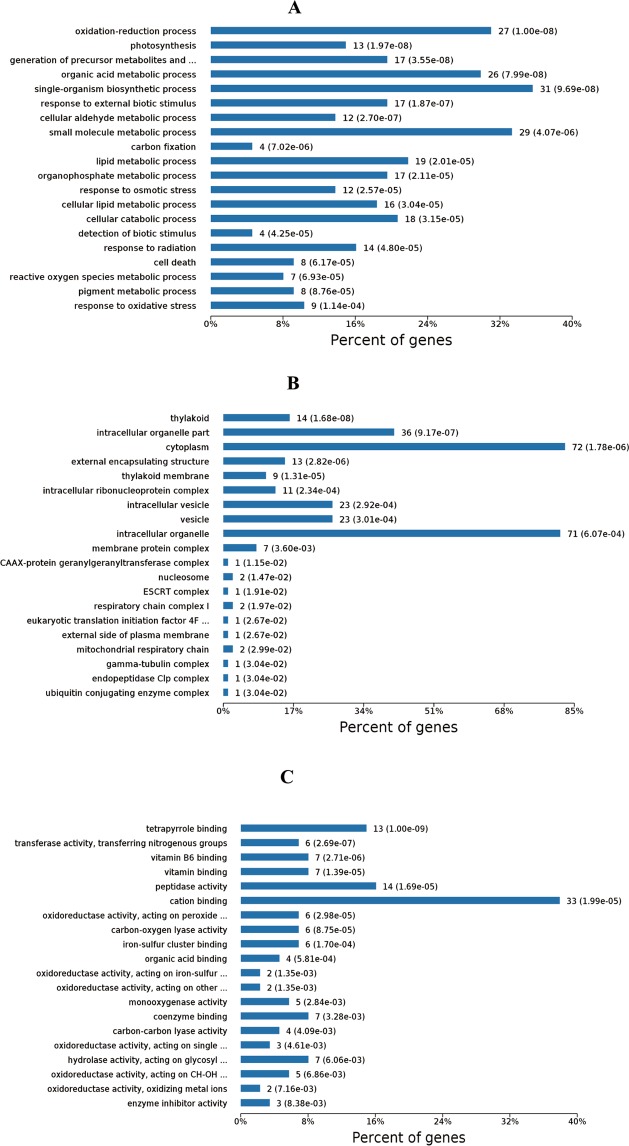
(**A**) The results of the biological processes with significant enrichment at Level 4. The horizontal axis represents the number of genes/proteins accumulated in each biological process. The P value is labeled after each bar. (**B**) The results of significant enrichment of cell components at Level 4. The horizontal axis represents the number of genes/proteins enriched by each cell component. The P value is labeled after each bar. (**C**) The results of molecular function enrichment at Level 4. The horizontal axis represents the number of genes/proteins enriched by each molecular function. The P value is labeled after each bar.

**Figure 2:**
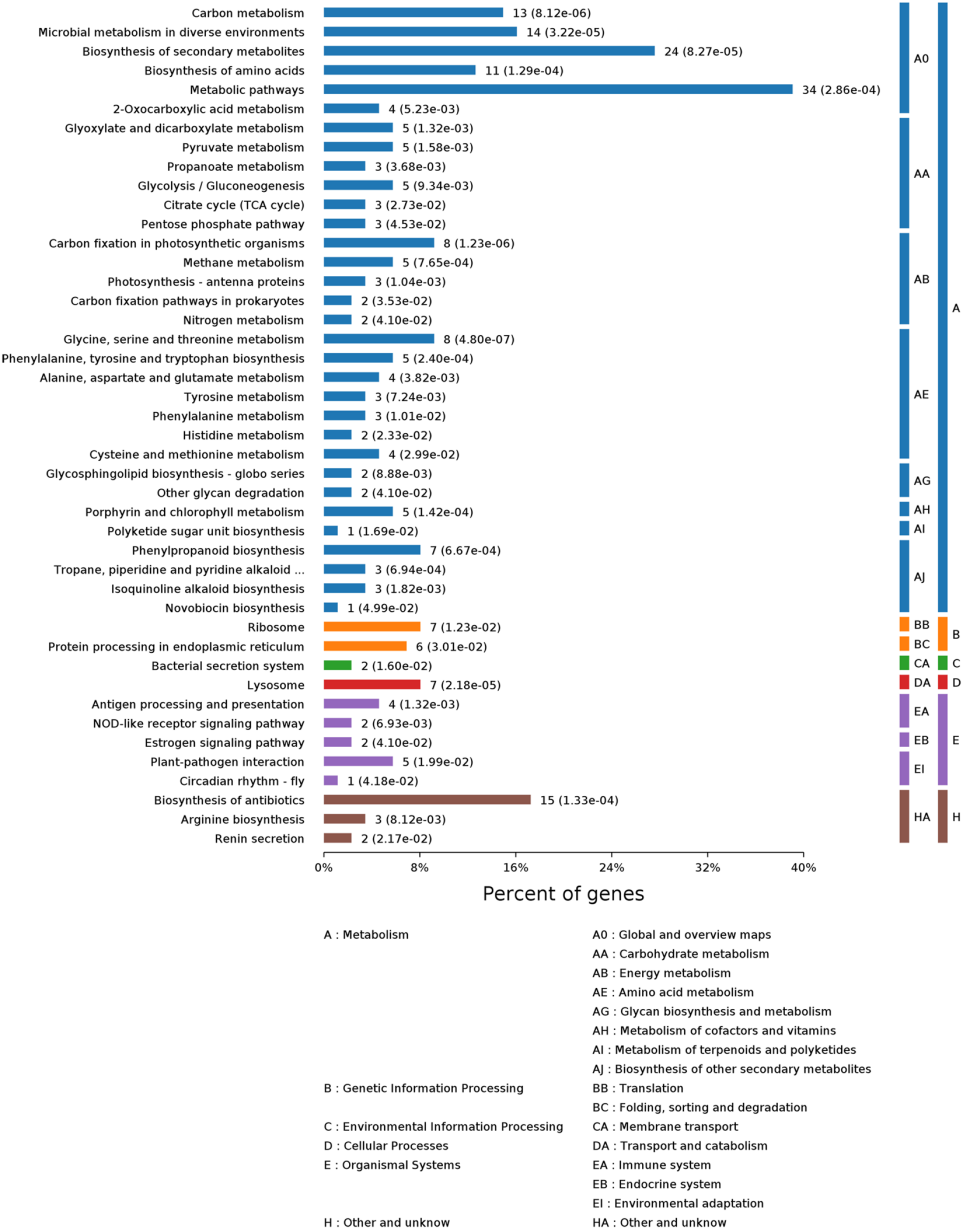
KEGG pathways were enriched at Level 4 between the treatment and control groups of hybrid bamboo. KEGG pathway classification indicating significantly enriched signaling pathways and their p values. The KEGG pathway is divided into the following categories in the KEGG database: (**A**) Metabolism, (**B**) Genetic Information Processing, (**C**) Environmental Information Processing, (**D**) Cellular Processes, (**E**) Organismal Systems, (**H**) Other and unknow.

**Figure 3:**
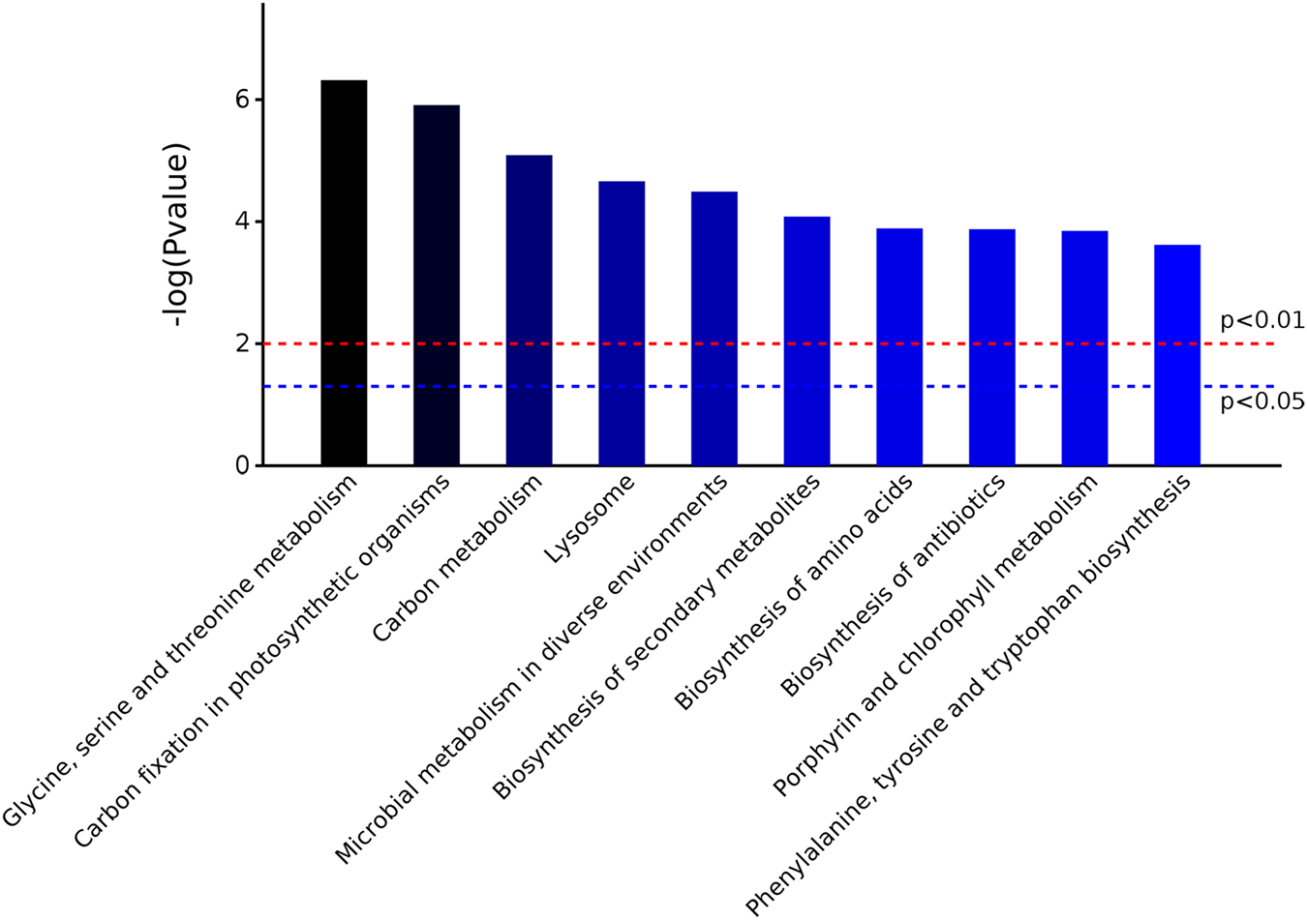
The top 10 KEGG signaling pathways with the most significant P value. The boundaries of P value 0.05 and 0.01 are marked.

The GO^28^ function of the identified hybrid bamboo proteins was analyzed according to three aspects: biological process, molecular function, and cell component under two treatment conditions: inoculation of *A. phaeospermum* suspension and sterile water inoculation. The results (Fig. 1) showed that the functional items (GO term) of protein enrichment in the cell component of hybrid bamboo was chloroplast, plastid part, chloroplast part, and chloroplast stroma. The functional items (GO term) where the proteins of hybrid bamboo were significantly enriched corresponded to molecular functions of oxidoreductase activity, tetrapyrrole binding, cofactor binding, and heme binding. The differentially expressed proteins of hybrid bamboo under different treatment conditions were significantly enriched in single-organism biosynthetic process (31 differentially expressed proteins) (Fig. 1A). The differentially expressed proteins of hybrid bamboo under different treatment conditions were significantly enriched in cytoplasm (72 differentially expressed proteins) and intracellular organelle (71 differentially expressed proteins) in the cell component (Fig. 1B). The differentially expressed proteins of hybrid bamboo under different treatment conditions were significantly enriched in cation binding (33 differentially expressed proteins) in the molecular function category (Fig. 1C). The differential proteins annotation in the GO database were shown in Table S2. KEGG^29^ enrichment analysis showed that the differential proteins of hybrid bamboo with the inoculation of *A. phaeospermum* and the sterile water were mainly concentrated in the metabolic pathways (Fig. 2). Among them, the metabolic pathways for the significant enrichment of differentially expressed proteins were metabolic pathways (map01100) (34 differentially expressed proteins), the biosynthesis of secondary metabolites (map01110) (24 differentially expressed proteins), microbial metabolism in diverse environments (map01120) (14 differentially expressed proteins), carbon metabolism (map01200) (13 differentially expressed proteins), and the biosynthesis of amino acids (map01230) (11 differentially expressed proteins) (Table 2). The differential proteins annotation in the KEGG database were shown in Table S3. The top 10 signaling pathways with the most significant P value in the KEGG enrichment analysis are shown in Fig. 3.

**Table 2:** Signaling pathway response to protein based on GO and KEGG analysis.

Terms	Count	P value	Protein IDs (Gene IDs)
**GO (gene ontology)**			
(BP)			
Single-organism metabolic process	31	8.99 × 10^−11^	PH01000011G2670|1.618939296;PH01000043G2150|1.543865433;P80089|1.874184585;UXS6|1.750945746;PH01000157G0430|1.585919766;CP12-2|1.799677343;CSY2|1.614675989;TSB2|1.898726748;PH01000290G0470|1.531403518;WAXY|1.672246023;At1g62810|1.982103184;PH01000742G0250|1.721182159;Q43495|5.480307181;PH01000898G0600|1.965363594;PH01001498G0080|1.765636566;P29305|2.211106384;NDB2|1.523793191;CYP72A15|2.085602899;PH01002248G0250|1.530194115;P80826|1.588424487;PER70|2.036694586;FER1|1.557846522;ASP1|1.721142225;P50477|1.572109245;PH01005469G0070|1.910034876;PH01005665G0070|1.648624898;Sb03g046810|1.723109056;PH01100083G0010|1.848329913;PAO|1.55076872;CAD5|1.611066513;VIT_19s0014g02480|2.117950844;LAC25|1.763531683;P83332|1.672999202;At1g74360|1.524451415;RPL9|0.575310035;PH01000024G2570|0.647692636;RPL24|0.571074195;PH01000137G0030|0.599065468;LPD1|0.565966308;NIR|0.650177682;CAP10A|0.501070054;UGT83A1|0.623960858;RPS10|0.618594113;PH01000761G0570|0.656611514;P29308|0.398024362;PH01001219G0020|0.611022423;PH01001303G0370|0.653209586;Q02066|0.592333828;PH01001720G0230|0.649297598;At4g16580|0.563926869;PH01002342G0040|0.616356462;PSRP2|0.627572934;PH01004347G0010|0.641820982
Small molecule metabolic process	29	4.07 × 10^−6^	PH01000011G2670|1.618939296;P80089|1.874184585;UXS6|1.750945746;CSY2|1.614675989;TSB2|1.898726748;PH01000290G0470|1.531403518;PH01000742G0250|1.721182159;Q43495|5.480307181;PH01001498G0080|1.765636566;P29305|2.211106384;PH01002248G0250|1.530194115;P80826|1.588424487;ASP1|1.721142225;P50477|1.572109245;PH01005469G0070|1.910034876;PH01005665G0070|1.648624898;PH01100083G0010|1.848329913;VIT_19s0014g02480|2.117950844;RPL9|0.575310035;RPL24|0.571074195;NIR|0.650177682;CAP10A|0.501070054;UGT83A1|0.623960858;RPS10|0.618594113;P29308|0.398024362;PH01001219G0020|0.611022423;Q02066|0.592333828;PH01001720G0230|0.649297598;PSRP2|0.627572934
**(CC)**			
Cytoplasm	72	1.78 × 10^−6^	PH01000011G2670|1.618939296;PH01000041G1710|2.682780333;ASPG1|1.508196275;P80089|1.874184585;PH01000121G1130|1.598740941;UXS6|1.750945746;PH01000157G0430|1.585919766;SD25|1.609550724;CP12-2|1.799677343;CSY2|1.614675989;TSB2|1.898726748;PH01000290G0470|1.531403518;WAXY|1.672246023;At1g62810|1.982103184;PATL3|1.665916623;BMY1|3.212227051;PH01000742G0250|1.721182159;Q43495|5.480307181;CYP-3|1.570124782;PH01000898G0600|1.965363594;PH01001131G0060|1.687345939;tlp|1.551620328;Zlp|1.673995861;PH01001498G0080|1.765636566;P29305|2.211106384;NDB2|1.523793191;CYP72A15|2.085602899;PH01002240G0140|1.500709346;PH01002248G0250|1.530194115;P80826|1.588424487;PER70|2.036694586;FER1|1.557846522;ASP1|1.721142225;P50477|1.572109245;PH01005469G0070|1.910034876;PH01005665G0070|1.648624898;Sb03g046810|1.723109056;PH01100083G0010|1.848329913;FTA|1.509636399;PAO|1.55076872;PRP40A|1.884300818;BETV1L|1.835519874;RPL29|1.754881288;Os09g0442300|1.930133039;CCP1|1.634896552;At1g74360|1.524451415;CATHB2|1.915507292;RPL9|0.575310035;PH01000052G1130|0.441589629;PH01000071G1780|0.58635762;RPL24|0.571074195;PH01000137G0030|0.599065468;LPD1|0.565966308;PH01000239G0580|0.662960971;NIR|0.650177682;At4g12770|0.59063367;CAP10A|0.501070054;RPS10|0.618594113;PH01000761G0570|0.656611514;P29308|0.398024362;PH01001219G0020|0.611022423;PH01001303G0370|0.653209586;TIM22-3|0.628426122;Q02066|0.592333828;PH01001720G0230|0.649297598;PH01002342G0040|0.616356462;RPS17|0.665658337;PSRP2|0.627572934;PH01004347G0010|0.641820982;Phyllostachys_edulis_newGene_36049|0.569207945;rps4|0.651115549;AGT1|0.595658203
Intracellular organelle	71	6.07 × 10^−4^	PH01000011G2670|1.618939296;PH01000041G1710|2.682780333;ASPG1|1.508196275;P80089|1.874184585;PH01000121G1130|1.598740941;UXS6|1.750945746;PH01000157G0430|1.585919766;SD25|1.609550724;CP12-2|1.799677343;CSY2|1.614675989;TSB2|1.898726748;PH01000290G0470|1.531403518;WAXY|1.672246023;At1g62810|1.982103184;PATL3|1.665916623;BMY1|3.212227051;PH01000742G0250|1.721182159;Q43495|5.480307181;CYP-3|1.570124782;PH01000898G0600|1.965363594;PH01001131G0060|1.687345939;tlp|1.551620328;Zlp|1.673995861;PH01001498G0080|1.765636566;P29305|2.211106384;NDB2|1.523793191;CYP72A15|2.085602899;PH01002240G0140|1.500709346;PH01002248G0250|1.530194115;P80826|1.588424487;PER70|2.036694586;FER1|1.557846522;ASP1|1.721142225;P50477|1.572109245;PH01005469G0070|1.910034876;PH01005665G0070|1.648624898;Sb03g046810|1.723109056;PH01100083G0010|1.848329913;PAO|1.55076872;PRP40A|1.884300818;BETV1L|1.835519874;RPL29|1.754881288;Os09g0442300|1.930133039;CCP1|1.634896552;At1g74360|1.524451415;CATHB2|1.915507292;RPL9|0.575310035;PH01000052G1130|0.441589629;PH01000071G1780|0.58635762;RPL24|0.571074195;PH01000137G0030|0.599065468;LPD1|0.565966308;PH01000239G0580|0.662960971;NIR|0.650177682;CAP10A|0.501070054;UGT83A1|0.623960858;RPS10|0.618594113;PH01000761G0570|0.656611514;P29308|0.398024362;PH01001219G0020|0.611022423;PH01001303G0370|0.653209586;TIM22-3|0.628426122;Q02066|0.592333828;PH01001720G0230|0.649297598;PH01002342G0040|0.616356462;RPS17|0.665658337;PSRP2|0.627572934;PH01004347G0010|0.641820982;Phyllostachys_edulis_newGene_36049|0.569207945;rps4|0.651115549;AGT1|0.595658203
**(MF)**			
Cation binding	33	1.99 × 10^−5^	PH01000011G2670|1.618939296;PH01000043G2150|1.543865433;P80089|1.874184585;PH01000157G0430|1.585919766;At1g62810|1.982103184;BMY1|3.212227051;Q43495|5.480307181;PH01000898G0600|1.965363594;P29305|2.211106384;NDB2|1.523793191;CYP72A15|2.085602899;PH01002248G0250|1.530194115;P80826|1.588424487;PER70|2.036694586;FER1|1.557846522;ASP1|1.721142225;P50477|1.572109245;PH01005469G0070|1.910034876;Sb03g046810|1.723109056;PH01100083G0010|1.848329913;PAO|1.55076872;CAD5|1.611066513;VIT_19s0014g02480|2.117950844;PAP24|1.676756578;LAC25|1.763531683;PH01000003G3180|0.613648731;PH01000137G0030|0.599065468;LPD1|0.565966308;NIR|0.650177682;CAP10A|0.501070054;PH01000761G0570|0.656611514;Q02066|0.592333828;PH01001720G0230|0.649297598
**Terms**	**Count**	**P value**	**Protein Names (Gene Names)**
**KEGG (Kyoto encyclopedia of genes and genomes) pathways**			
Metabolic pathways	34	2.86 × 10^−4^	PH01000011G2670|1.618939296;PH01000041G1710|2.682780333;PH01000043G2150|1.543865433;UXS6|1.750945746;CSY2|1.614675989;TSB2|1.898726748;PH01000290G0470|1.531403518;At1g62810|1.982103184;Q43495|5.480307181;PH01000898G0600|1.965363594;PH01001498G0080|1.765636566;P29305|2.211106384;PH01002248G0250|1.530194115;P80826|1.588424487;PER70|2.036694586;ASP1|1.721142225;P50477|1.572109245;PH01005469G0070|1.910034876;PH01005665G0070|1.648624898;Sb03g046810|1.723109056;PH01100083G0010|1.848329913;CAD5|1.611066513;VIT_19s0014g02480|2.117950844;PH01000071G1780|0.58635762;PH01000137G0030|0.599065468;LPD1|0.565966308;CAP10A|0.501070054;PH01000761G0570|0.656611514;P29308|0.398024362;PH01001303G0370|0.653209586;Q02066|0.592333828;PH01001720G0230|0.649297598;PH01002342G0040|0.616356462;AGT1|0.595658203
Biosynthesis of secondary metabolites	24	8.27 × 10^−5^	PH01000041G1710|2.682780333;PH01000043G2150|1.543865433;PH01000157G0430|1.585919766;CSY2|1.614675989;TSB2|1.898726748;At1g62810|1.982103184;PH01000898G0600|1.965363594;P29305|2.211106384;P80826|1.588424487;PER70|2.036694586;ASP1|1.721142225;PH01005469G0070|1.910034876;PH01005665G0070|1.648624898;Sb03g046810|1.723109056;PH01100083G0010|1.848329913;PAO|1.55076872;CAD5|1.611066513;LPD1|0.565966308;PH01000761G0570|0.656611514;P29308|0.398024362;PH01001303G0370|0.653209586;Q02066|0.592333828;PH01002342G0040|0.616356462;AGT1|0.595658203
Microbial metabolism in diverse environments	14	3.22 × 10^−5^	PH01000011G2670|1.618939296;CSY2|1.614675989;PH01000290G0470|1.531403518;P29305|2.211106384;PH01002248G0250|1.530194115;P80826|1.588424487;ASP1|1.721142225;PH01005665G0070|1.648624898;PH01000137G0030|0.599065468;LPD1|0.565966308;NIR|0.650177682;P29308|0.398024362;Q02066|0.592333828;AGT1|0.595658203
Carbon metabolism	13	8.12 × 10^−6^	PH01000011G2670|1.618939296;CSY2|1.614675989;PH01000290G0470|1.531403518;P29305|2.211106384;PH01002248G0250|1.530194115;P80826|1.588424487;ASP1|1.721142225;PH01005665G0070|1.648624898;PH01000137G0030|0.599065468;LPD1|0.565966308;P29308|0.398024362;Q02066|0.592333828;AGT1|0.595658203
Biosynthesis of amino acids	11	1.29 × 10^−4^	PH01000041G1710|2.682780333;CSY2|1.614675989;TSB2|1.898726748;PH01000290G0470|1.531403518;P29305|2.211106384;P80826|1.588424487;ASP1|1.721142225;PH01005469G0070|1.910034876;PH01005665G0070|1.648624898;P29308|0.398024362;Q02066|0.592333828

### PPI Analysis

**Figure 4:**
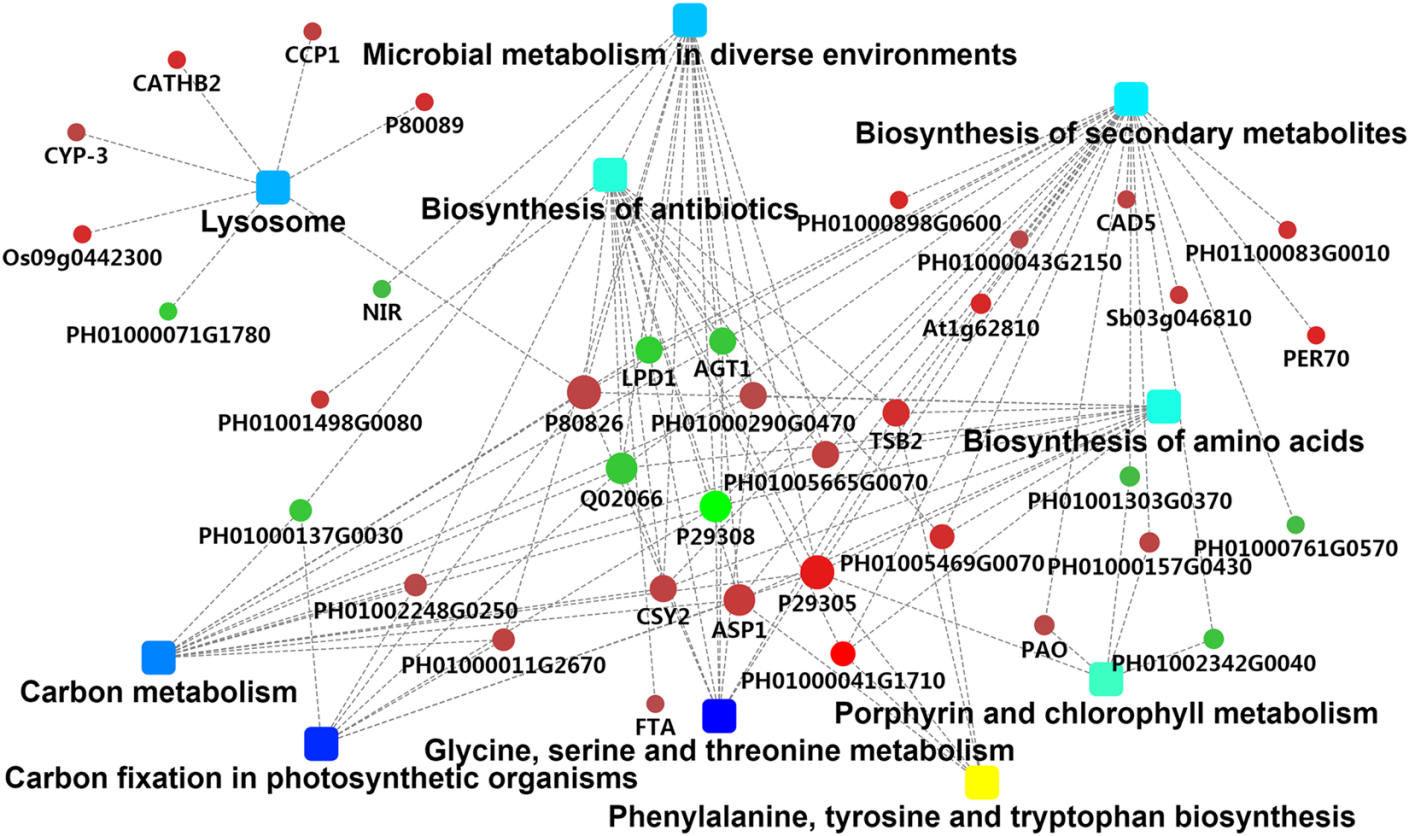
PPI network map of differential proteins in the treatment and control groups of hybrid bamboo.(It was drawn by using Cytoscape software. Its version was 3.7.1 and URL linkwas cytoscape.org. Round node represents the protein/gene (for fold change analysis, red indicates upregulation, green indicates downregulation), rectangular node represents the KEGG pathway/biological process, significant p value is expressed by the yellow–blue gradient, yellow indicates the p value is smaller, blue indicates the p value is larger.

The PPI analysis chart (Fig. 4) was drawn by using Cytoscape software (version 3.7.1. URL link: cytoscape.org.), based on the gene-interaction network that incorporates protein-protein interaction results along with other pieces of evidence like fold-change, KEGG term enrichment, and biological process enrichment. The most prominently upregulated proteins with high connectivity between the treatment group and the control group are shown in red, and metabolic pathways in dark blue. Among them, D-isomer specific 2-hydroxyacid dehydrogenase, catalytic domain (ID: PH01000290G0470), Transaldolase (ID:PH01005665G0070), Indole-3-glycerol phosphate synthase (ID: PH01005469G0070), and Glutamine amidotransferase class-I, Peptidase C26 (ID: PH01000041G1710) were upregulated, and participated in several significantly enriched metabolic pathways through other significant protein/gene interactions in the treatment group and control group of hybrid bamboo (Table 3).

**Table 3:** High connectivity differential protein information in PPI.

Protein ID	Description	KEGG pathways
PH01000290G0470		Lysosome
		Carbon metabolism
		Microbial metabolism in diverse environments
		Biosynthesis of antibiotics
		Glycine, serine, and threonine metabolism
		Phenylalanine, tyrosine, and tryptophan biosynthesis
		Biosynthesis of amino acids
PH01005665G0070		Lysosome
		Carbon metabolism
		Biosynthesis of antibiotics
		Microbial metabolism in diverse environments
		Biosynthesis of secondary metabolites
		Biosynthesis of amino acids
PH01005469G0070		Biosynthesis of antibiotics
		Biosynthesis of secondary metabolites
		Biosynthesis of amino acids
		Phenylalanine, tyrosine, and tryptophan biosynthesis
PH01000041G1710		Biosynthesis of antibiotics
		Biosynthesis of secondary metabolites
		Biosynthesis of amino acids
		Phenylalanine, tyrosine, and tryptophan biosynthesis

### LC-PRM/MS statistical analysis and PRM validation of 11 differential proteins

In order to verify the accuracy of TMT and LC-MS/MS data analysis, LC–PRM/MS was used to analyze the candidate peptides of the 11 target proteins (These 11 peptides are unique peptides of each protein), basing on the up-regulated and down-regulated differentially expressed proteins in the GO and KEGG databases, and combing with functional annotation information. The Skyline analysis results of each peptides are shown Fig. S1 and include the chromatographic peak contrast map (Skyline analysis map). After the Skyline analysis of 3–5 sub-ions with high abundance and as continuous as possible in the secondary mass spectrometry, the peak area of each target peptide (Table S4) was obtained. The ionic peak area of the corresponding peptide is presented in Table S5. The results of quantitative analysis of target peptide fragments in different samples are shown in Table S6. The quantitative results of PRM (Table 4) showed that 10 of the 11 candidate proteins were similar to the trend of TMT. They showed a fold change > 1.2 times with a significance of less than 0.05. The trend consistency between PRM validation and TMT data was 91% demonstrating that the data obtained by TMT combined with LC-MS/MS in this experiment were reliable. Among them, there results for differentially expressed proteins Pyruvate phosphate dikinase (ID:PH01000011G2670), Alcohol dehydrogenase GroES-like domain (ID:PH01000043G2150), Ribulose bisphosphate carboxylase (ID: PH01000137G0030), Peroxidase (ID: PH01000761G0570, PH01000898G0600), Clp protease (ID: PH01001219G0020), DnaJ domain (ID: PH01002240G0140), Indole-3-glycerol phosphate synthase (ID: PH01005469G0070), and PEP/pyruvate binding domain (ID: PH01002248G0250) (ID: PH01100083G0010)were similar to those obtained with TMT. All proteome PRM data of 2 samples of hybrid bamboo was deposited in the integrated proteome resources under the accession number, respectively.

**Table 4:** Quantitative information table of 11 candidate proteins by PRM and TMT.

Protein ID	Protein Name	Peptide Sequence	Treatment (*A. phaeospermum* Inoculation)	Control (sterile water)	Fold ChangeTMT	Fold Change PRM	Consistent with the quantitative trend of TMT data
PH01000011G2670	Pyruvate phosphate dikinase, PEP/pyruvate binding domain; PEP-utilising enzyme	TPEDLDAMR	18800935.74	6482210.915	1.62	2.90	Yes
PH01000043G2150	Alcohol dehydrogenase GroES-like domain	ANVEQYCNK	9216705.267	2813699.21	2.68	3.28	Yes
PH01000137G0030	Ribulose bisphosphate carboxylase, Ribulose-1,5-bisphosphate carboxylase small subunit	ANVEQYCNK	95884188.96	112125351.9	0.60	0.86	Yes
PH01000742G0250	—	DAEGAGIYGSQGR	8081460.326	10726002.86	1.72	0.75	No
PH01000761G0570	Peroxidase	NNPSDIDPSLNPSYAK	1347740.717	2437134.947	0.66	0.55	Yes
PH01000898G0600	Peroxidase	MGNINPLTGTAGQIR	605242794	183946954.6	1.97	3.29	Yes
PH01001219G0020	Clp protease	SSSSYSQHR	1356342.289	1881885.675	0.61	0.72	Yes
PH01002240G0140	HSCB C-terminal oligomerisation domain; DnaJ domain	EAVNEASDSQTLEK	4627480.999	1873152.026	1.50	2.47	Yes
PH01002248G0250	PEP-utilising enzyme, Pyruvate phosphate dikinase, PEP/pyruvate binding domain; PEP-utilising enzyme,	ELCSETGADQEDALAR	14857013.63	4716898.7	1.53	3.15	Yes
PH01005469G0070	Indole-3-glycerol phosphate synthase	DIEEELGAPR	2008165.237	1062576.02	1.91	1.89	Yes
PH01100083G0010	—	YFSAAASQALDTAER	14580393.99	3450921.436	1.85	4.23	Yes

## Discussion

TMT-labeled nano-liquid chromatography tandem mass spectrometry (LC-MS/MS), a new quantitative method technology, overcomes the shortcomings of traditional methods which can not quantify macromolecules and proteins^30^. This is the first application in the proteome study of hybrid bamboo under the stress of the pathogenic fungus *A. phaeospermum*. Plants evolve complex disease resistance mechanisms in their struggle against pathogenic fungi^31^. The expression of proteins related to plant disease resistance will change to complete signal sensing and transmission, which will lead to plant response mechanism. The formation of disease resistance in all plants is closely related to the change of protein quantity and function. Differential proteomics focuses on finding differences in protein expression between samples caused by specific factors, which can reveal changes in plant proteomes under specific physiological or pathological conditions. At present, the proteomics of plants infected by various pathogenic fungi have been studied. For example, *Arabidopsis thaliana* under the stress of *Fusarium*, the rice under the stress of *Magnaporthe grisea*^32,33^, the wheat under the stress of *Fusarium graminearum*^34,35^, the barley under *Gibberella scab* stress^36^, *Brassica carinata* under the stress of *Phytophthora parasitica*^37^, the cauliflower under *Xanthomonas campestris* stress^38^, the alfalfa under *Aphanomyces euteiches* stress were studied, and the differential proteomes were obtained and identified^39–41^. The expression of resistance-related proteins in those plants increased more or less. Combined the results of this study with the above studies, the expression of proteins related to disease resistance in hybrid bamboo infected by pathogenic fungus *A. phaeospermum* may increase, comparing with that in hybrid bamboo inoculated with sterile water. The results indicated that under the stress of the pathogenic fungi, the expression of proteins related to self-resistance would inevitably be affected during the growth of hybrid bamboo. This study will help to identify the key proteins of plant disease resistance, so as to formulate new strategies for disease prevention and control.

On the other hand, 102 differentially expressed proteins were identified by TMT-labeled quantitative proteomics from hybrid bamboo treated with sterile water and *A.phaeospermum*, of which 66 were up-regulated and 36 were down-regulated. PRM targeted quantitative proteomics combined with LC-PRM/MS quantitative analysis was used to quantitatively analyze 11 differential candidate proteins. The TMT results of differential proteins Pyruvate phosphate dikinase (ID:PH01000011G2670), Alcohol dehydrogenase GroES-like domain (ID:PH01000043G2150), Peroxidase (ID:PH01000898G0600, PH01000761G0570), DnaJ domain (ID:PH01002240G0140), PEP/pyruvate binding domain (ID:PH01002248G0250), Indole-3-glycerol phosphate synthase (ID:PH01005469G0070), (ID: PH01100083G0010), Ribulose bisphosphate carboxylase (ID:PH01000137G0030), Clp protease (ID:PH01001219G0020) were consistent with the quantitative results of PRM. Bioinformatics analysis showed that the differentially up-regulated proteins of hybrid bamboo infected by pathogenic fungi were significantly enriched in the biological processes, cell components and molecular functions of GO database, compared with those of hybrid bamboo inoculated with sterile water. Differential proteins are mainly concentrated in monomer metabolism, redox and carboxylic acid metabolism. The cell components are mainly enriched in chloroplast and plastid components, and the molecular functions are mainly enriched in oxidoreductase activity, tetrapyrrole binding and heme binding. Previous studies have shown that the redox processes of wheat, *Ligustrum lucidum*, locust, jujube, poplar and sugar beet seedlings under drought stress are all affected, and the reactive oxygen species (ROS) in the redox process have the role of signal molecules, which can regulate programmed cell death^42–45^. Chloroplast is the site of plant photosynthesis, which can produce a variety of reactive oxygen species^46^. It is suggested that there are a large number of proteins involved in physiological and biochemical processes such as redox in hybrid bamboo under the pathogenic fungi stress, which may be related to plant stress resistance. There were 102 differential proteins were annotated to different pathways in KEGG metabolic pathway enrichment analysis. These pathways included secondary metabolites synthesis (map01110), microbial metabolism in different environments (map01120), carbon metabolism (map01200) and amino acid biosynthesis (map01230). Previous studies have indicated that plant secondary metabolites such as phytoprotegerin, lignin and peroxidase can be used as biochemical barriers to resist pathogenic bacteria invasion and participate in signal transduction of plant disease resistance^47–49^. In addition, many substances in secondary metabolites, including flavonoids, flavonoids, ginkgolides and phenolics, play an important role in plant growth and development, physiological and biochemical metabolism, stress resistance and disease resistance^50,51^. For example, the content of isoflavones in alfalfa leaves increased after infected with *Phytophthora* spp^52^. Based on the above conclusions, the differential proteins of hybrid bamboo infected by the pathogenic fungus *A. phaeospermum* were significantly enriched in the synthesis of secondary metabolites. Because the cell wall degrading enzymes and debris produced by cell wall degradation may trigger the defense response of plants themselves, it can be inferred that the cell wall degrading enzymes are secreted by *A. phaeospermum*. Secreted proteins may cause changes in the expression of related proteins in hybrid bamboo in response to this process^53^. Among them, cinnamyl alcohol dehydrogenase in the up-regulation protein of hybrid bamboo infected by *A. phaeospermum* plays a key role in plant lignin synthesis, which can provide necessary strength, hydrophobicity and resistance to external pathogenic fungi^54–56^. Furthermore, the up-regulated protein cysteine protease belonging to the protease family causes programmed cell death in plant tissues under stress to resist pathogenic fungi infection^57^. Therefore, it is speculated that the differential proteins of hybrid bamboo inoculated with the pathogenic fungus *A. phaeospermum* are significantly enriched in redox process, secondary metabolite synthesis and amino acid synthesis, and there are proteins related to disease resistance of hybrid bamboo. At present, only the metabolic pathways of candidate disease-resistant proteins in hybrid bamboo have been studied, but the specific functions of these proteins need to be further verified.

## Materials and Methods

### Materials

Plant materials: one-year-old hybrid bamboo plants were planted in the bamboo-growing areas of a reclaimed farmland (103°01′N, 29°54′E) in Sichuan, China. The study area was at an altitude of 515.98 m with an annual temperature of 6.8 to 26.1 °C, and annual precipitation of 1300–1700 mm. Hybrid rice was planted in the rice-growing areas of Chengdu Plain (103°01′N, 29°54′E) in Sichuan, China. The area has an altitude of 530 meters, average annual temperature of 15.9 °C, and average annual precipitation of 1010 mm. All samples were healthy varieties.

Microorganism: *A. phaeospermum* was isolated from diseased hybrid bamboo^23^. The isolate was maintained on a PDA slant medium containing potato dextrose agar at 4 °C until used.

Instruments and reagents: UA buffer (8 M urea, 150 mM Tris–HCl pH 8.0), NH_4_HCO_3_ (Sigma, A6141), acetonitrile (Merck, 1499230-935), TMT 10plex kit (Thermo Fisher), Pierce High PH Reversed-Phase Peptide Fractionation Kit (Thermo Fisher), Q-Exactive Plus (Thermo Scientific), Easy-nLC1200 (Thermo Scientific), Trap column (Reverse-phase), 100 μm × 20 mm (5 μm, C18), Thermo Scientific EASY column (Reverse-phase), 75 μm × 120 mm (3 μm, C18), MaxQuant (version 1.6.0.16).

### Sample preparation

Thirty one-year-old hybrid bamboo plants were selected. Fifteen annual hybrid bamboos were inoculated with *A. phaeospermum* suspension using the needling method^21^. And the other fifteen annual hybrid bamboos were inoculated with sterile water as a control. Each plant was inoculated with eight tender branches (needling at the tip of the tender branch fork, not piercing), and the injection volume was about 50 μL at the wound. Bagging was conducted to kept them wet for 12 hours and inoculation was repeated for 3 consecutive days. When the hybrid bamboo showed symptoms of shoot blight, samples were taken from the tender shoots of hybrid bamboo that had been treated with a suspension of *A. phaeospermum* and sterile water, respectively. Three replicates per treatment group. Samples were frozen in liquid nitrogen and stored at −80 °C until use.

### Protein sample preparation

Three buds of hybrid bamboo inoculated with *A. phaeospermum* and three buds of hybrid bamboo inoculated with sterile water were ground into powder by liquid nitrogen. Pyrolysis solution (200 μL) was added to 30 mg of powder, followed by ultrasonic treatment and TCA–acetone precipitation at −20 °C overnight. Then, samples were centrifuged for 15 minutes at 4 °C, 16,000 *g*, washed, and precipitated twice with cold acetone, and dried in air. Then, 150 μL pyrolysis solution was added to each sample in a tube, and the sample was centrifuged for 16,000 g for 15 min. The supernatant was retained and the protein concentration was determined by BCA^58^ (Beyotime Institute of Biotechnology, Shanghai, China). The protein concentration was calculated by measuring the absorbance at 562 nm. SDS-PAGE gel electrophoresis: 20 μg protein samples 5:1 (v/v) were taken from each group and added into 5 × sample buffer solution. The samples were bathed in boiling water for 5 min. SDS-PAGE (8%–16%) electrophoresis was carried out followed by Coomassie brilliant blue staining.

### Enzymatic hydrolysis of proteins, desalination, and quantification

A total of 300 μg of each sample was taken for enzymatic hydrolysis^59^, DTT was added to 100 mM, followed by boiling in a water bath for 5 min and cooling to room temperature. A total of 200 μL UA buffer (8 M urea, 150 mM Tris–HCl pH 8.0) was added to the mix, then transferred into a 10 kDa ultrafiltration centrifuge tube, centrifuged for 12,000 *g* for 15 min, before adding 200 μL UA buffer. This mixture was centrifuged again at 12,000 *g* for 15 min, and the filtrate was discarded. We then added 100 μL IAA (Iodoacetamide) (50 mM IAA in UA), followed by oscillation at 600 rpm for 1 min, before incubation at room temperature for 30 min and centrifugation at 12,000 *g* for 10 min. Remove the filtrate. Then, we added 100 μL UA buffer, centrifuged for 12,000 *g* for 10 min and this process was repeated twice. Remove the filtrate. Following this, NH_4_HCO_3_ buffer was added, and the mixture was then centrifuged at 14,000 *g* for 10 min, and this process was repeated twice. Remove the filtrate. A 40 μL aliquot of trypsin buffer (6 μg trypsin in 40 μL NH_4_HCO_3_ buffer) was added, followed by oscillation at 600 rpm for 1 min, and then incubation at 37 °C for 16–18 hours in a new collection tube. This was then centrifuged at 12,000 *g* for 10 min, followed by the collection of filtrate, to which 0.1% TFA solution was added. A C18 cartridge was desalinated to quantify the peptide.

### TMT Labeling of Peptides and Peptide fractionation

Peptides (100 μg) were taken from each sample and labeled with the TMT 6-plex labeling kit according to manufacturer’s specifications. The labeled peptide fragments were evenly mixed, and the dried peptide fragments were separated by a Pierce High PH Reversed-Phase Peptide Fractionation Kit (Thermo Fisher). Finally, the samples were collected and merged into 10 frations. The peptides of each component were dried and then re-dissolved with 0.1% FA for LC–MS analysis.

### LC–MS/MS analysis of re-soluble peptide solution

LC–MS/MS analysis was carried out^10,60^. Peptides of each fraction were injected once, and 10 runs of mass spectrometric analyses were conducted. The sample components were separated by the liquid phase system Easy nLC for high-performance liquid chromatography. The chromatographic column was balanced by 95% A solution (0.1% formic acid solution). Samples were added to the chromatographic column, which was a trap column (2 cm × 100 μm, 5 μm C18). A Thermo Scientific EASY column (75 μm × 100 μm, 3 μm C18) was used for separation with a flow rate of 300 nL/min. The correlation gradient was as follows: the linear gradient of the B solution (0.1% acetonitrile formate solution) ranged from 4% to 7% in 0–2 min; at 2–57 min, the linear gradient of the B liquid ranged from 7% to 30%; at 57–62 min, the linear gradient of the B liquid ranged from 30% to 45%; at 62–67 min, the linear gradient of the B liquid ranged from 45% to 90%; and at 67–75 min, solution B was maintained at 90%.

The peptide fragments were separated by chromatography and analyzed by a Q-Exactive Plus mass spectrometer (Thermo Science) for 75 minutes. The scanning range of the parent ions was 300–1800 *m/z* with positive ions as a detection mode. The mass–charge ratios of polypeptide and polypeptide fragments were collected according to the following methods. Twenty debris maps (MS2 scan, HCD) were collected after each full scan. First-order mass spectrometry resolution: 70,000 @ *m/z* 200, AGC target: 1e6, first-order maximum IT: 50 ms. Resolution of secondary mass spectrometry: 17,500 @ *m/z* 200, AGC target: 1e5, secondary maximum IT: 50 ms, MS2 activation type: HCD, isolation window: 1.6 Th, normalized collision energy: 35.

### Data analysis

The mass spectrometry proteomics data have been deposited to the ProteomeXchange Consortium (http://proteomecentral.proteomexchange.org) via the iProX partner repository^61^ with the dataset identifier PXD014363, the subject ID is IPX0001646002. The final LC–MS/MS original RAW file was imported into MaxQuant^62,63^ software (version number 1.6.0.16) for database retrieval. In this experiment, the bamboo transcriptome translated protein database was selected for retrieval and analysis. The main database lookup parameter settings are shown in Table 5. Decoy and True protein sequence databases (Decoy is the reverse of the real database, in theory, its number is the same as the real database, but the sequence is wrong) were used for protein retrieval. The search results would get False Protein (FP, from Decoy DB) and True Protein (TP, from True DB). We controlled data quality through FDR (False Discovery Rate). FDR is the ratio of FP to the sum of FP and TP. FDR was set to be less than 0.01, that was, if we found 100 proteins, the error rate of controlling it was less than 1.

**Table 5:** MaxQuant search library parameter settings.

Item	Value
Type	Reporter ion MS2
Isobaric labels	TMT 6plex
Enzyme	Trypsin
Reporter mass tolerance	0.005 Da
Max Missed Cleavages	2
Main search Peptide Tolerance	4.5 ppm
First search Peptide Tolerance	20 ppm
MS/MS Tolerance	20 ppm
Fixed modifications	Carbamidomethyl (C)
Variable modifications	Oxidation (M), Acetyl (Protein N-term),
Database	Bamboo.fasta
Database pattern	Target-Reverse
PSM FDR^a^	≤0.01
Protein FDR	≤0.01
Protein quantification	Razor and unique peptides were used for protein quantification.

^a^FDR = FP/ (FP + TP), FP (False Protein, from Decoy database) and TP (True Protein, from True database).

### Bioinformatic analysis of proteome data

Bioinformatic analysis was carried out on the data of hybrid bamboo inoculated with *A. phaeospermum* and with sterile water. The GO database program of Blast2GO (http://geneontology.org/)^28^ was used to annotate the functions of proteins. The KEGG database (http://www.genome.jp/kegg/)^29^ was used to classify and group the identified proteins, and the Fisher’s Exact Test was used to analyze and calculate the significance level of each pathway and a GO term protein enrichment. Gene ontology (GO) functional analysis of the target protein and KEGG pathway analysis were carried out. The protein–protein interaction (PPI) data were obtained by introducing the protein gene into the IntAct molecular interaction database for retrieval. The results were downloaded in the XGMML format and imported into the Cytoscape software package to visualize and further analyze the functions of differential proteins. In addition, the importance of each protein in the PPI network was evaluated by calculating the degree of interaction between each protein.

### LC–PRM/MS analysis

According to the analysis results of the original labeled quantitative proteomics project, 11 candidate peptide of target protein were selected for targeted shotgun mass spectrometry analysis (Table 4). The unique peptides were screened by existing only in the target protein, of which the length is suitable for mass spectrometry analysis. There were no dynamic modification sites in the peptides, and a symmetrical and sharp chromatographic peak must be formed in LC-MS analysis. Finally, it was determined that each peptide of each target protein had reliable identification information (Fig. S2), which could be used for PRM quantitative analysis. The information of the peptide suitable for PRM analysis was imported into the software Xcalibur^64^ (version 4.0. Thermo Scientific) to set up the PRM method. The mass spectrometry proteomics data have been deposited to the Proteome Xchange Consortium (http://proteomecentral.proteomexchange.org) via the iProX partner repository^61^ with the dataset identifier PXD014364, the subject ID is IPX0001651003. The PRM method in the software Xcalibur was used to analyze the information of the peptides. The samples were analyzed by LC–PRM/MS using 2 μL of sample, the analysis was carried out using an Easy nLC1200 chromatographic system (Thermo Scientific). The chromatographic column was balanced with 95% liquid A (0.1% formic acid aqueous solution). The sample was added to the trap column (100 μm × 20 mm, 5 μm C18, Dr Maisch GmbH). Gradient separation was carried out on a chromatographic column (75 μm × 150 mm, 3 μm C18, Dr. Maisch GmbH) at a flow rate of 300 nL/min. The gradient of liquid phase separation was as follows:

0–5 min: The linear gradient of solution B ranged from 2% to 5%.

5–45 min: The linear gradient of solution B ranged from 5% to 23%.

45–50 min: The linear gradient of solution B was from 23% to 40%.

50–52 min: The linear gradient of solution B ranged from 40% to 100%.

52–60 min: solution B maintained at 100%.

Targeted mass spectrometry was performed with Q-Exactive Plus mass spectrometer (Thermo Scientific) after the peptides were separated. The analysis time was 75 minutes and positive ion was used as the detection method. The scanning range of the parent ions was 350–1500 *m/z*, the resolution of first-order mass spectrometry was 70000 @ *m/z* 200, the AGC target was 3e6, and the first-order maximum IT was 200 ms. Secondary mass spectrometry analysis of the peptide was conducted according to the following methods. After each full scan, 11 target peptides were selected according to the inclusion list for secondary mass spectrometry analysis (MS2). Skyline software and theoretical enzymolysis protein database (peptide) were used to screen, to ensure that only the unique peptide in the protein can be used for subsequent PRM analysis. The specific process was to carry out the theoretical digestion of the target protein, and the peptide sequence obtained was compared with the theoretical peptide of the whole protein database, and those peptides that belonged to and only belonged to the target protein were selected for PRM quantitative analysis. The MS2 resolution: 17,500 @ *m/z* 200; AGC target: 3e6; secondary mass spectrometry maximum IT: 100 ms; MS2 activation type: HCD; isolation window: 2.0 Th; and normalized collision energy: 27. The original RAW files of mass spectrometry were analyzed by PRM data using Skyline^65^ 4.1 software.

LC–PRM/MS analysis of candidate peptides of the target protein can obtain chromatographic peak contrast maps of each peptide in different samples, and quantitative analysis of 3–5 sub-ions with high abundance and as continuous as possible in the secondary mass spectrometry of candidate peptides can obtain a peak area of each target peptide. Based on the full scan information obtained from the original mass spectrometry data of each sample, the average ion peak intensity (Average Basepeak Intensity) of each sample was extracted by software RawMeat (Version 2.1, VAST Scientific)^16^. The normalization factor for each sample was calculated according to the average strength of the ion peak. The formula for calculating the normalization factor is as follows:

*f*_*N*_ of sample N = average ionic peak strength of all samples/average ionic peak strength of sample N.

By multiplying the ion peak area of each candidate peptide in sample N by the normalization coefficient *f*_*N*_, the ion peak area of the corresponding peptide normalized in sample N can be obtained. The normalized peak area of the peptide was used to quantitatively analyze the target peptide in different samples. Based on the quantitative information of each candidate peptide segment, the quantitative information of the target protein could be calculated by averaging the ratio of the peptides.

## Supplementary information

Supplementary Information 1
