# Supplementary material for: Differential Proteome Analysis of Hybrid Bamboo (Bambusa pervariabilis × Dendrocalamopsis grandis) Under Fungal Stress (Arthrinium phaeospermum)
Source: Sci Rep. 2019 Dec 10;9:18681. doi: 10.1038/s41598-019-55229-0 (PMC6904554; doi:10.1038/s41598-019-55229-0)

Figure S1 Skyline analysis of candidate peptide fragments of target proteins in different samples

PH01000011G2670 TPEDLDAMR

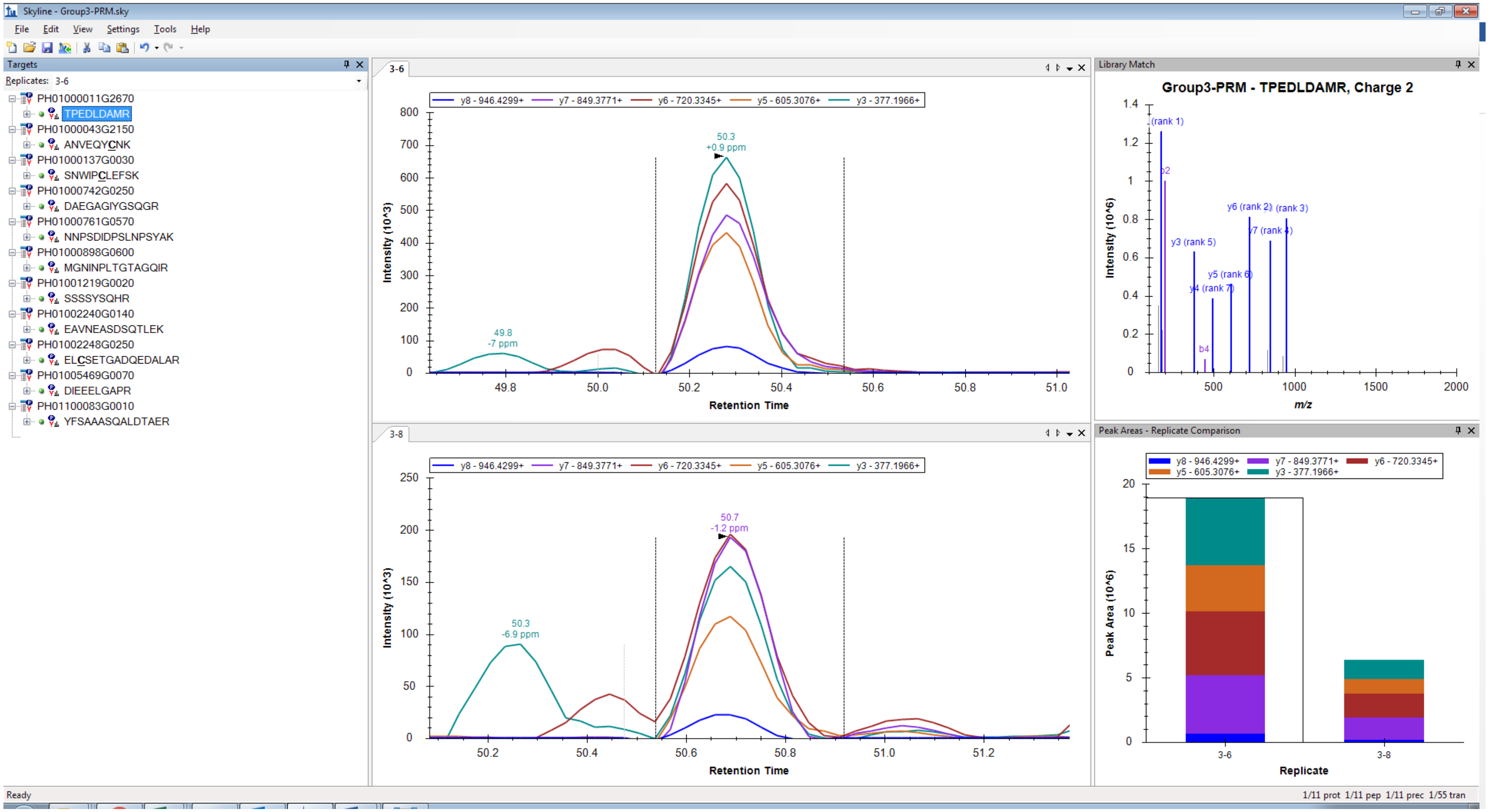

PH01000043G2150   ANVEQYCNK

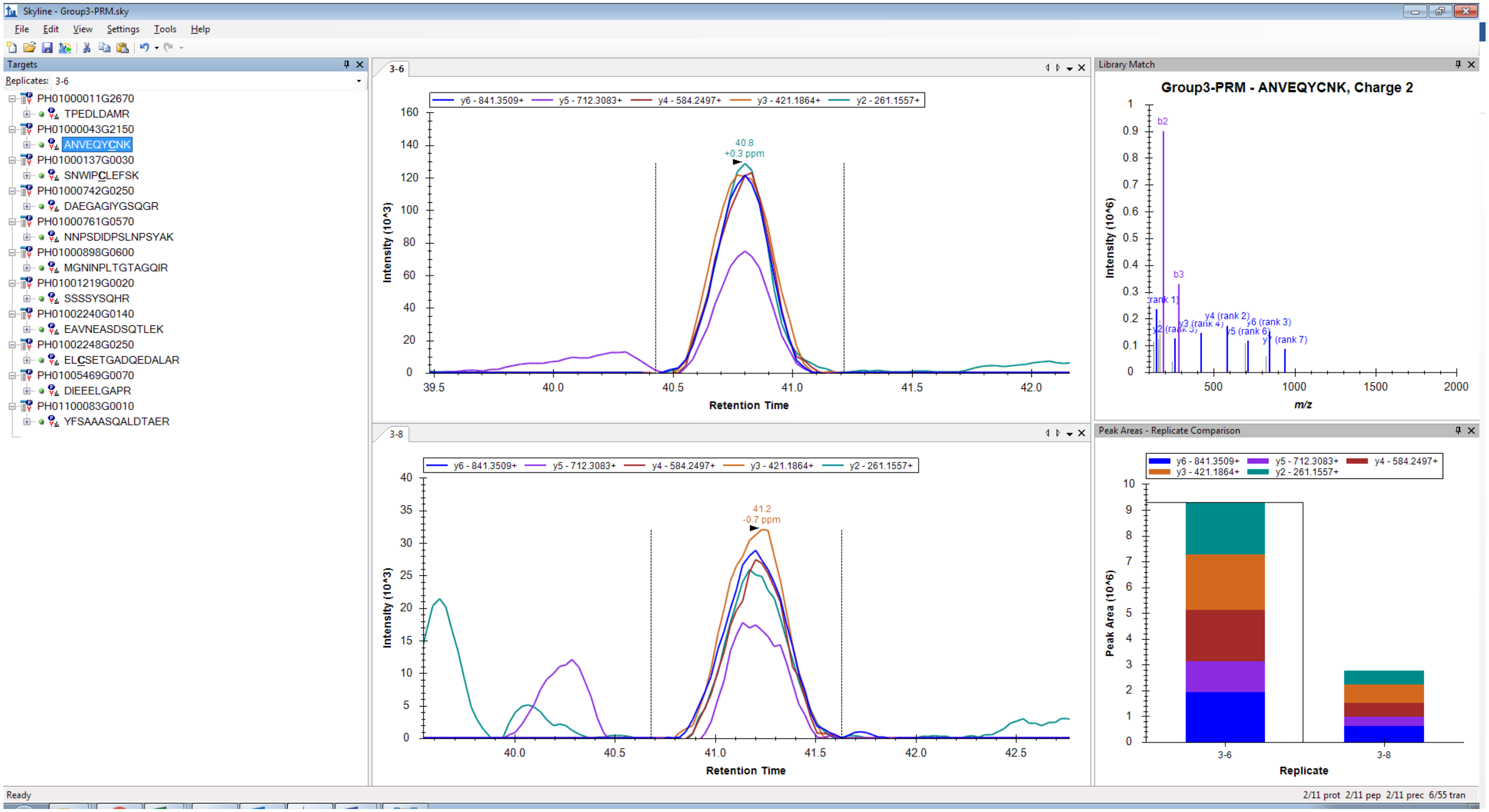

PH01000137G0030 SNWIPCLEFSK

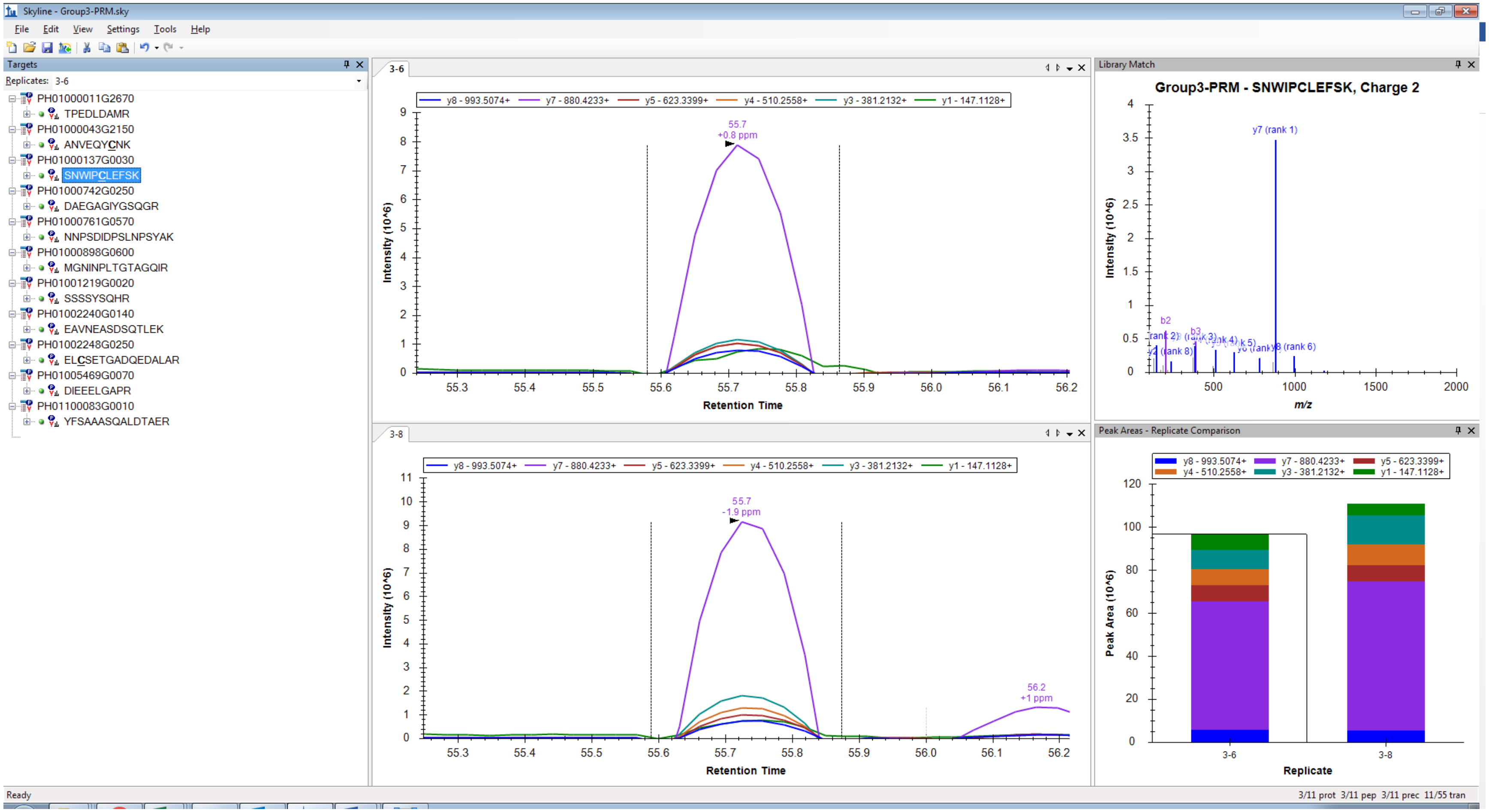

PH01000742G0250 DAEGAGIYGSQGR

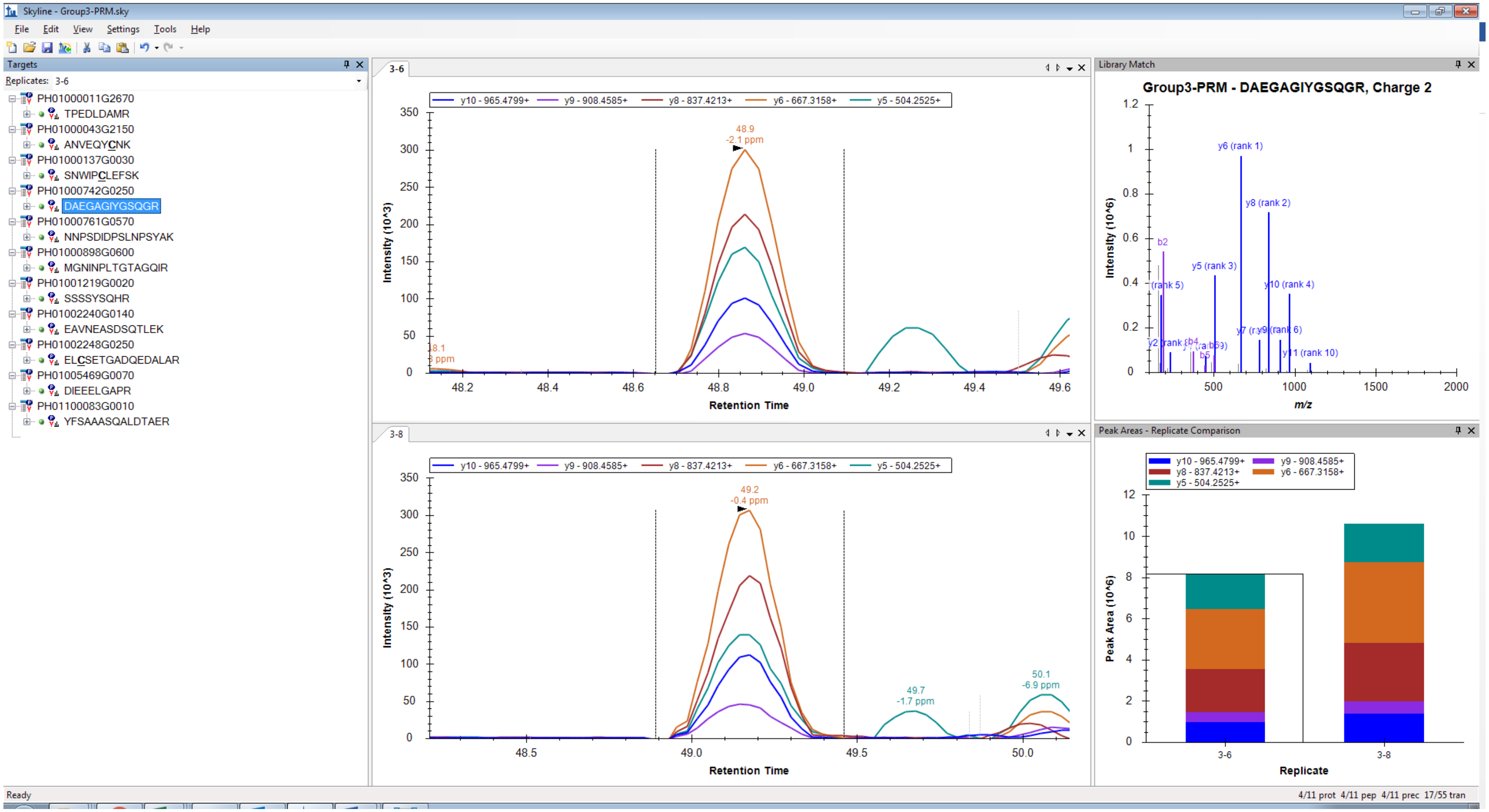

PH01000761G0570 NNPSDIDPSLNPSYAK

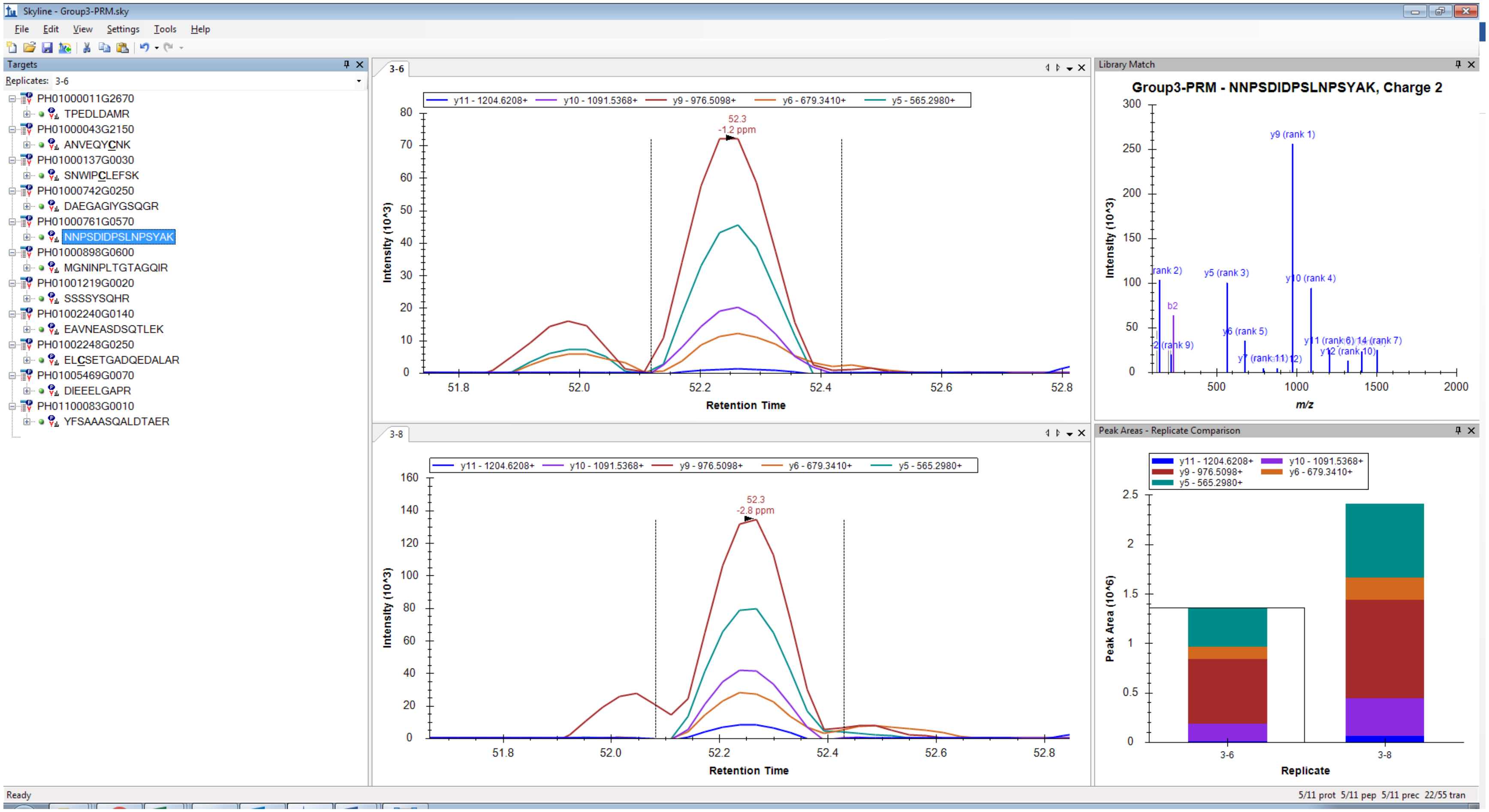

PH01000898G0600 MGNINPLTGTAGQIR

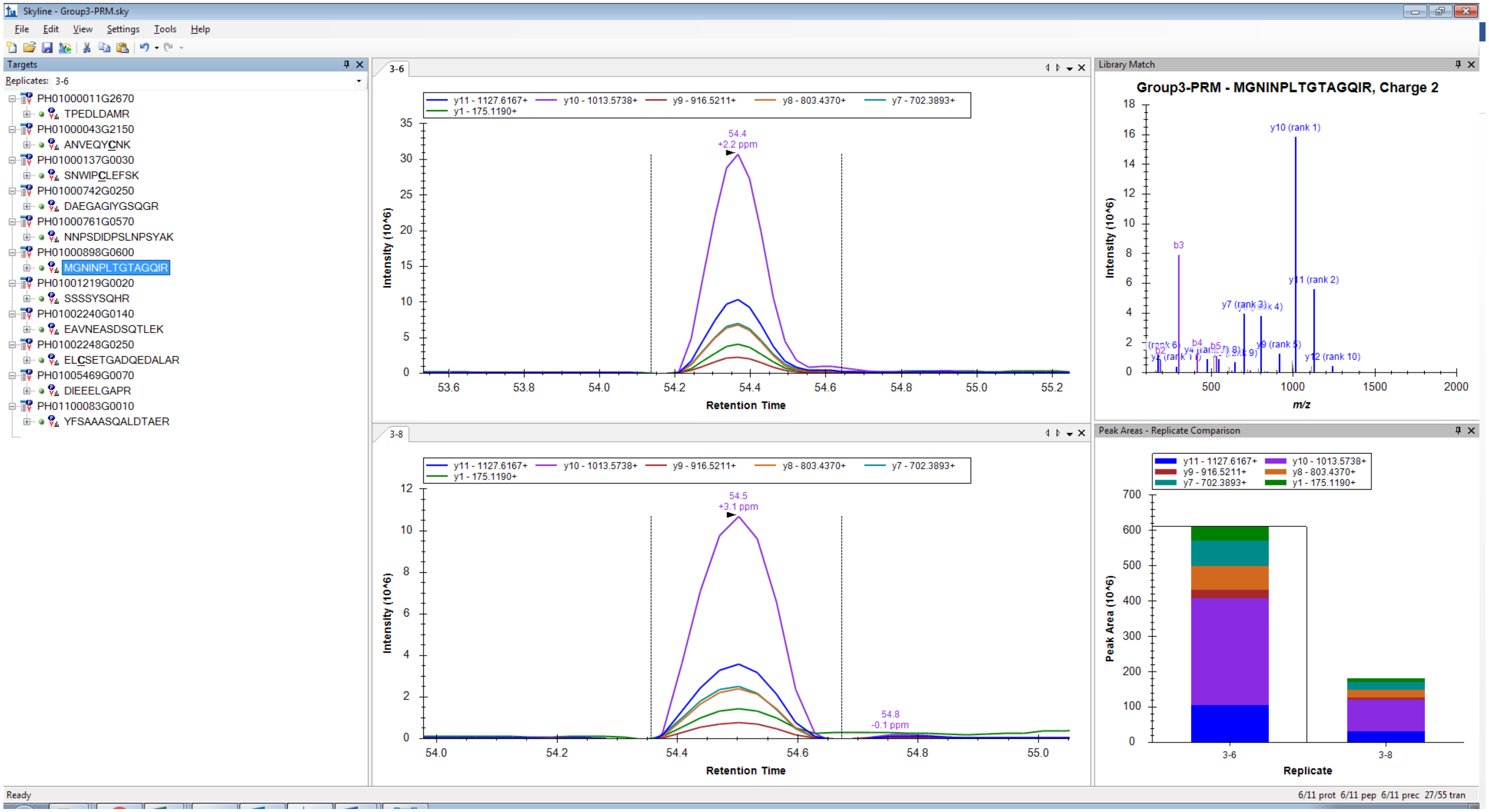

PH01001219G0020 SSSSYSQHR

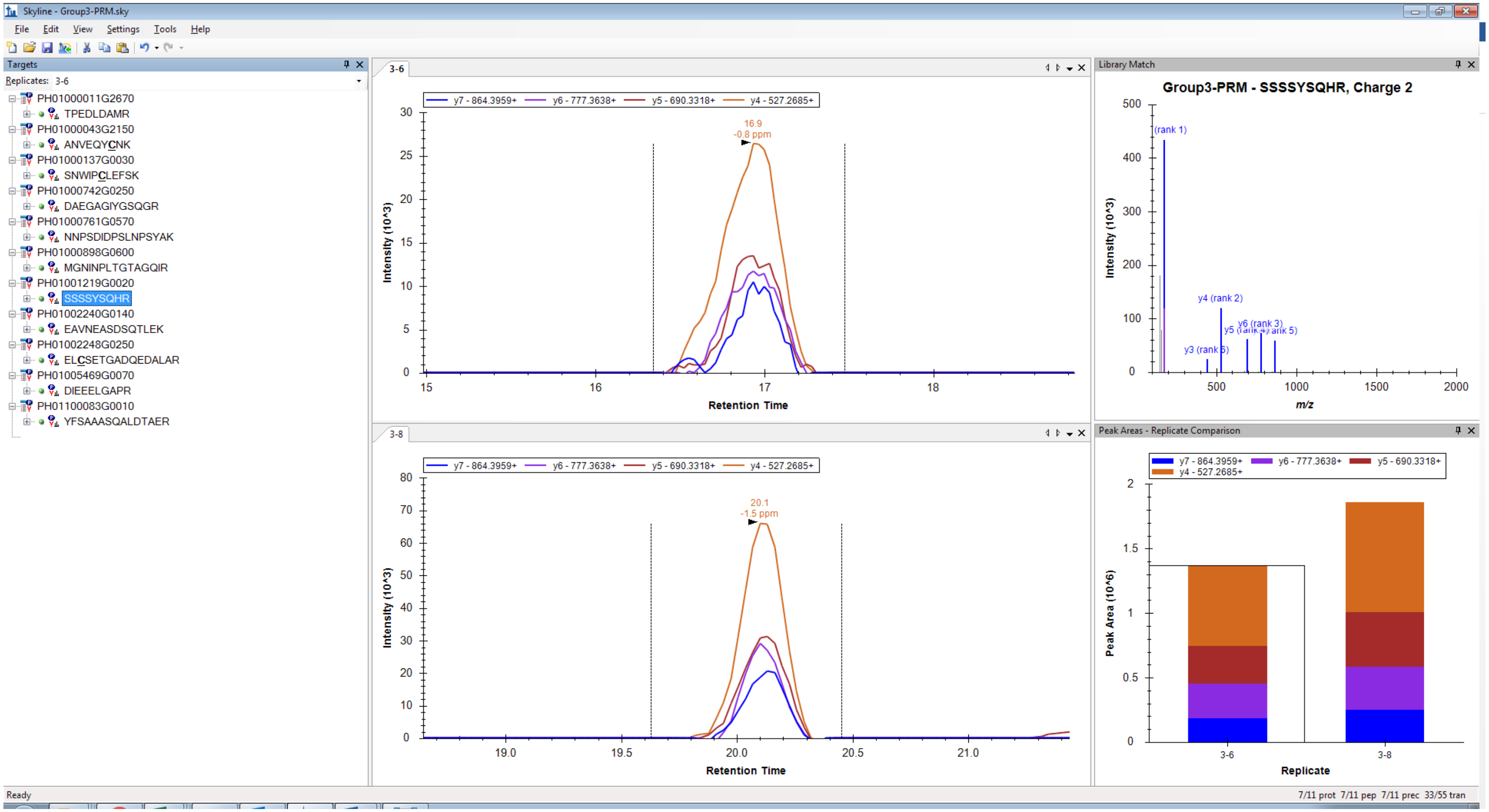

PH01002240G0140 EAVNEASDSQTLEK

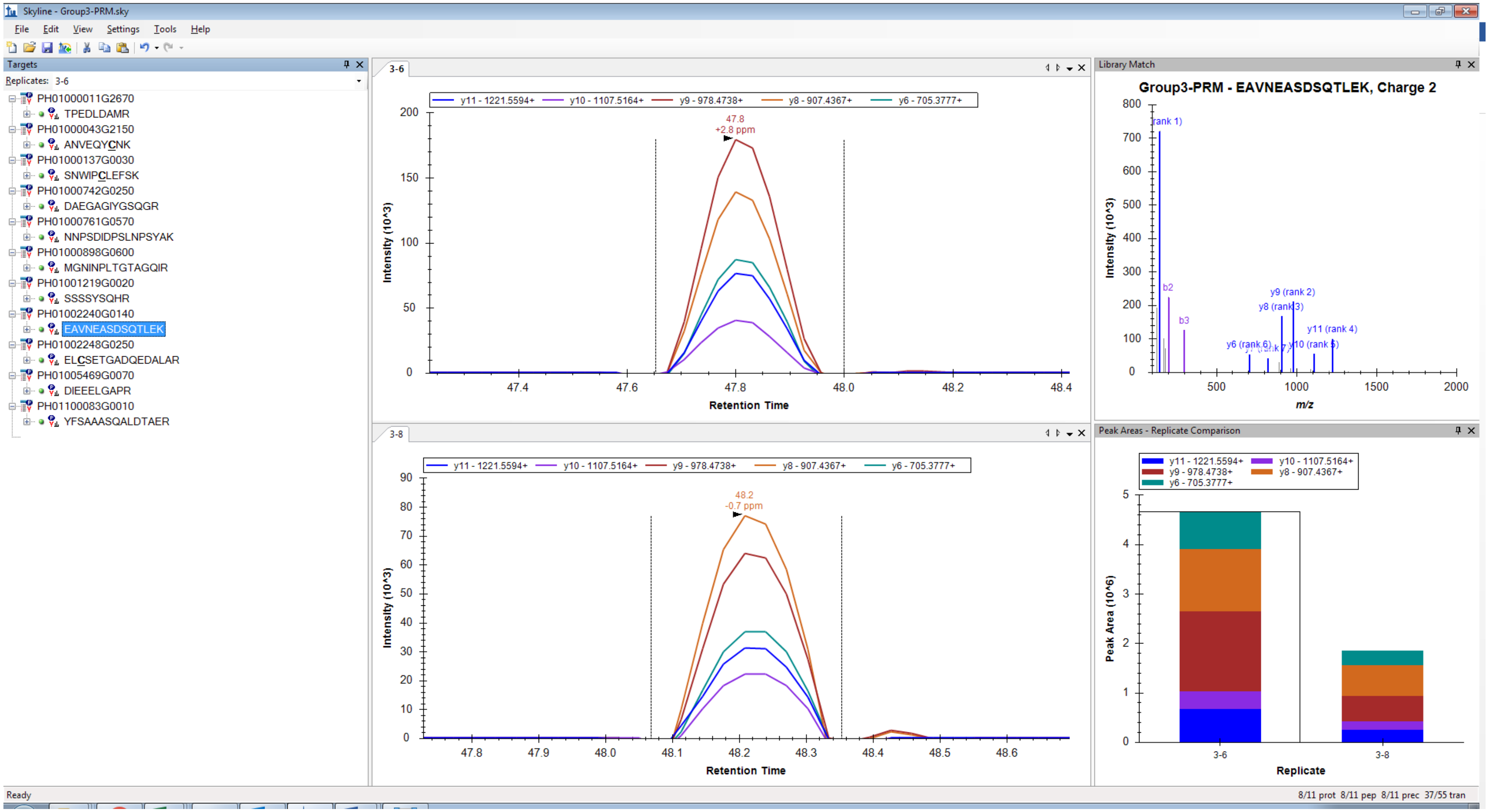

PH01002248G0250   ELCSETGADQEDALAR

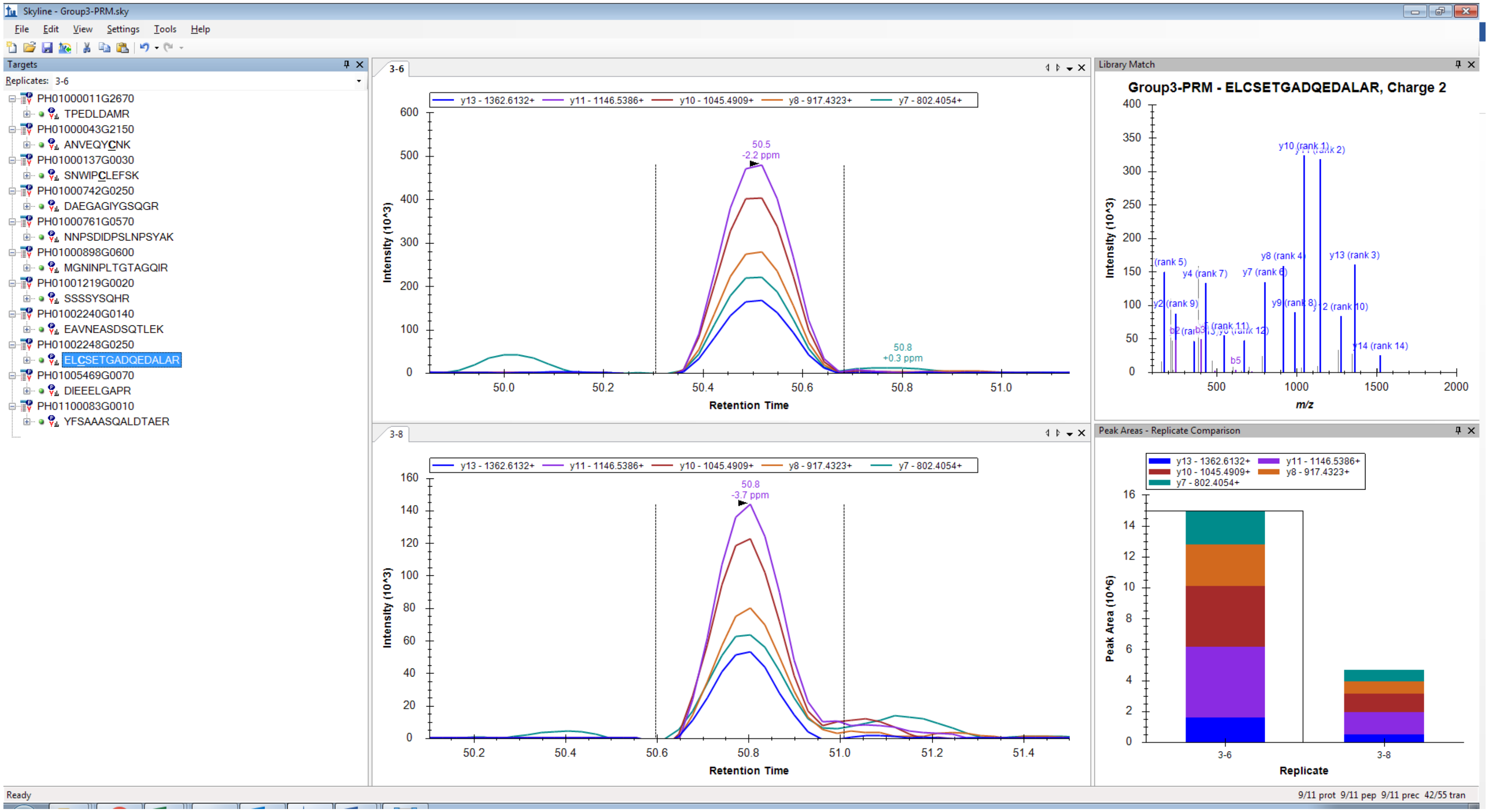

PH01005469G0070 DIEEELGAPR

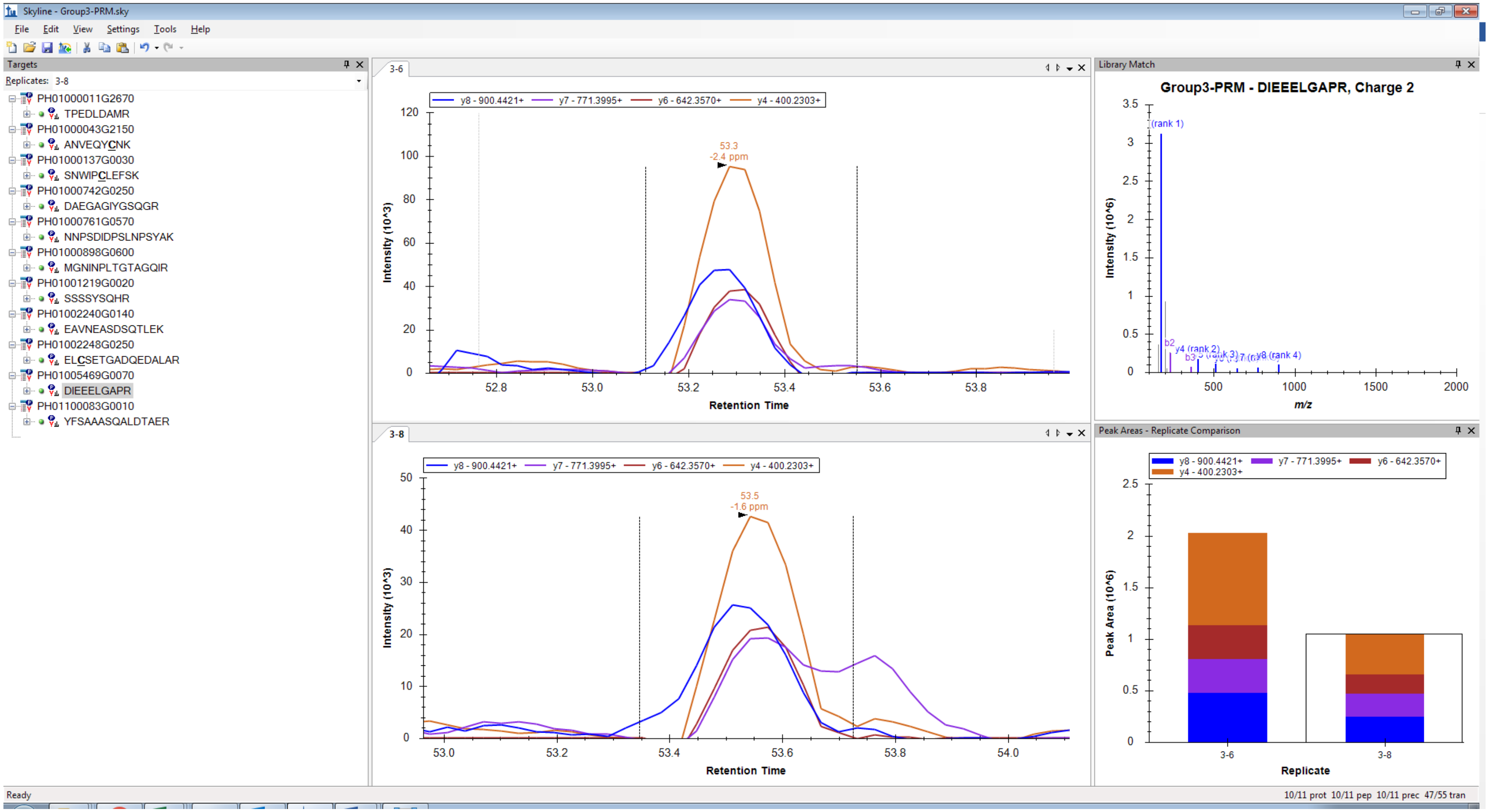

PH01100083G0010   YFSAAASQALDTAER

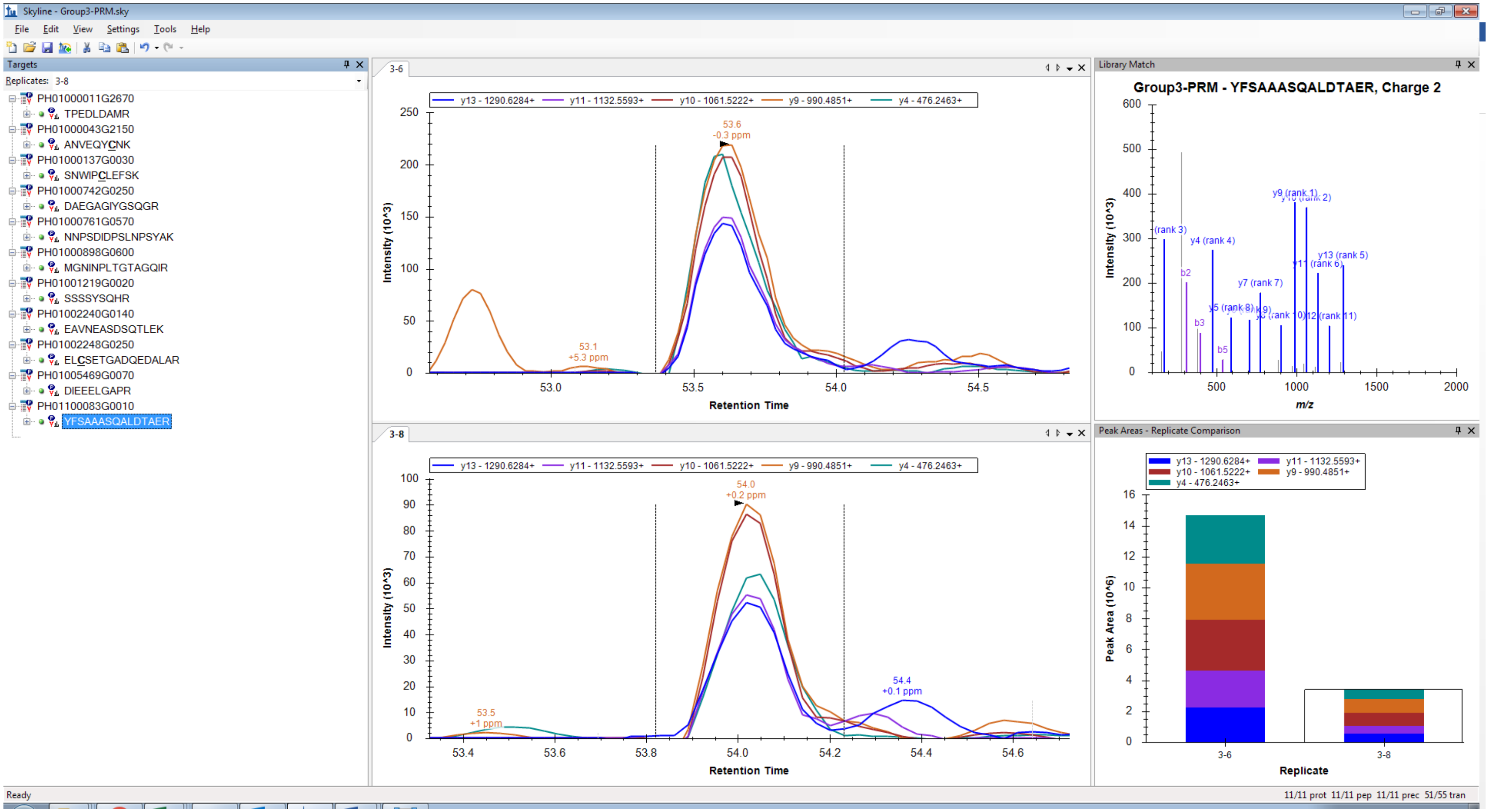

Supplement: Supplementary file 1 — Supplementary Information 1 [file 41598_2019_55229_MOESM1_ESM.zip › Supplementary materials/Figure S1 Skyline analysis of candidate peptide fragments of target proteins in different samples.pdf]
