# Supplementary material for: Differential Proteome Analysis of Hybrid Bamboo (Bambusa pervariabilis × Dendrocalamopsis grandis) Under Fungal Stress (Arthrinium phaeospermum)
Source: Sci Rep. 2019 Dec 10;9:18681. doi: 10.1038/s41598-019-55229-0 (PMC6904554; doi:10.1038/s41598-019-55229-0)
Supplement: Supplementary file 1 — Supplementary Information 1 [file 41598_2019_55229_MOESM1_ESM.zip › Supplementary materials/Figure S2 Secondary mass spectrometric matching map of candidate peptide segments.pdf]

Figure S2 Secondary mass spectrometric matching map of candidate peptide segments

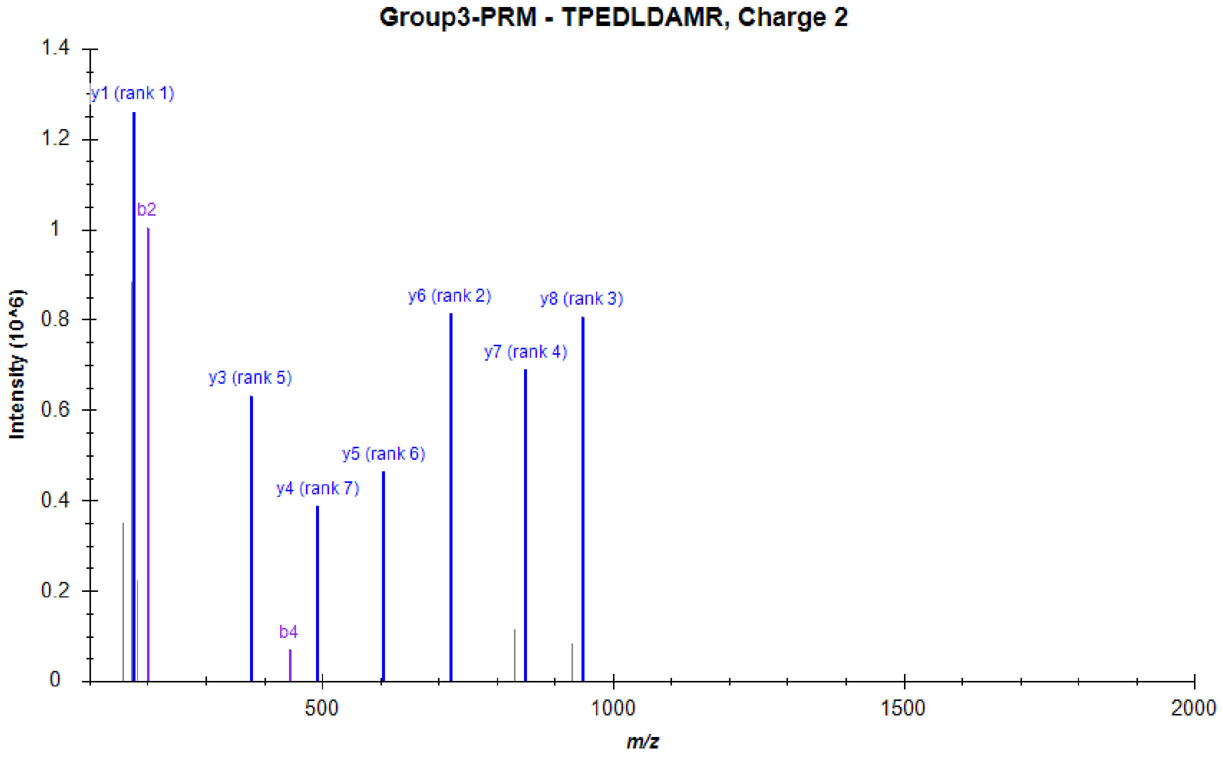

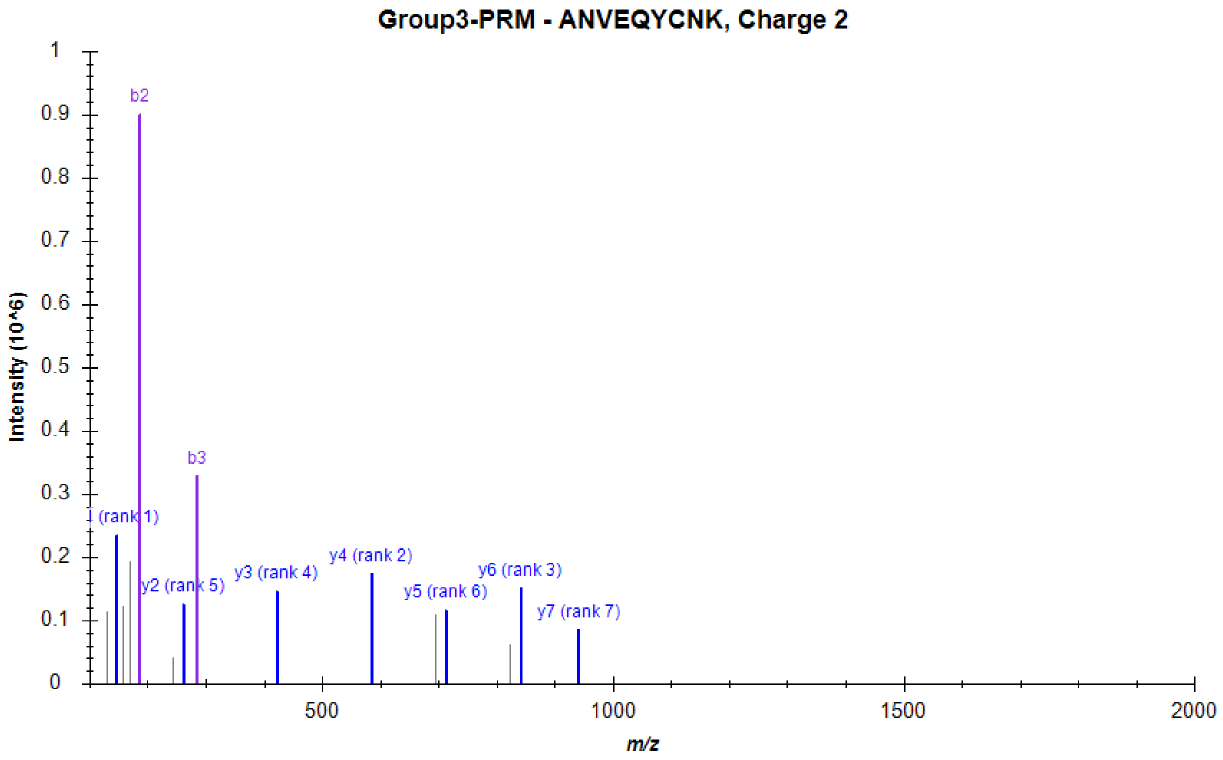

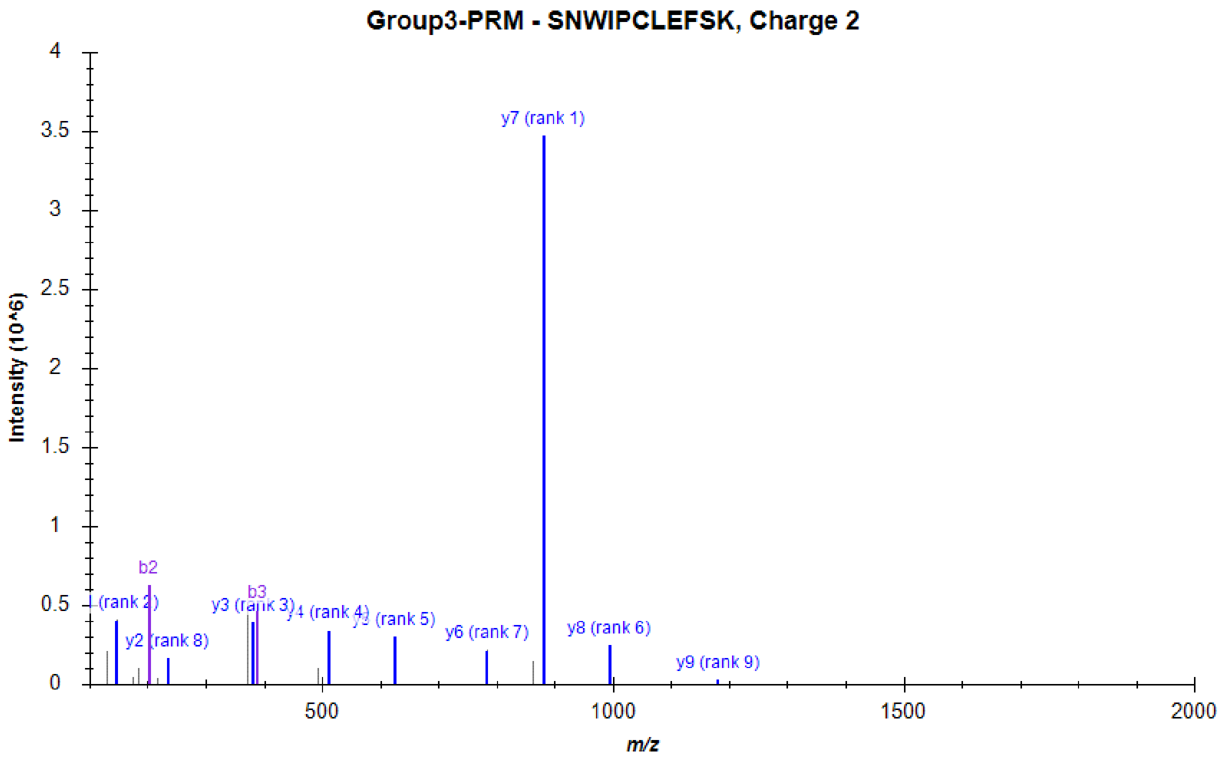
Group3-PRM - SNWIPCLEFSK, Charge 2
Intensity (10^6)
m/z
y7 (rank 1)
b2
b3
y2 (rank 8)
y3 (rank 3)
y6 (rank 7)
y8 (rank 6)
y9 (rank 9)
(rank 5)

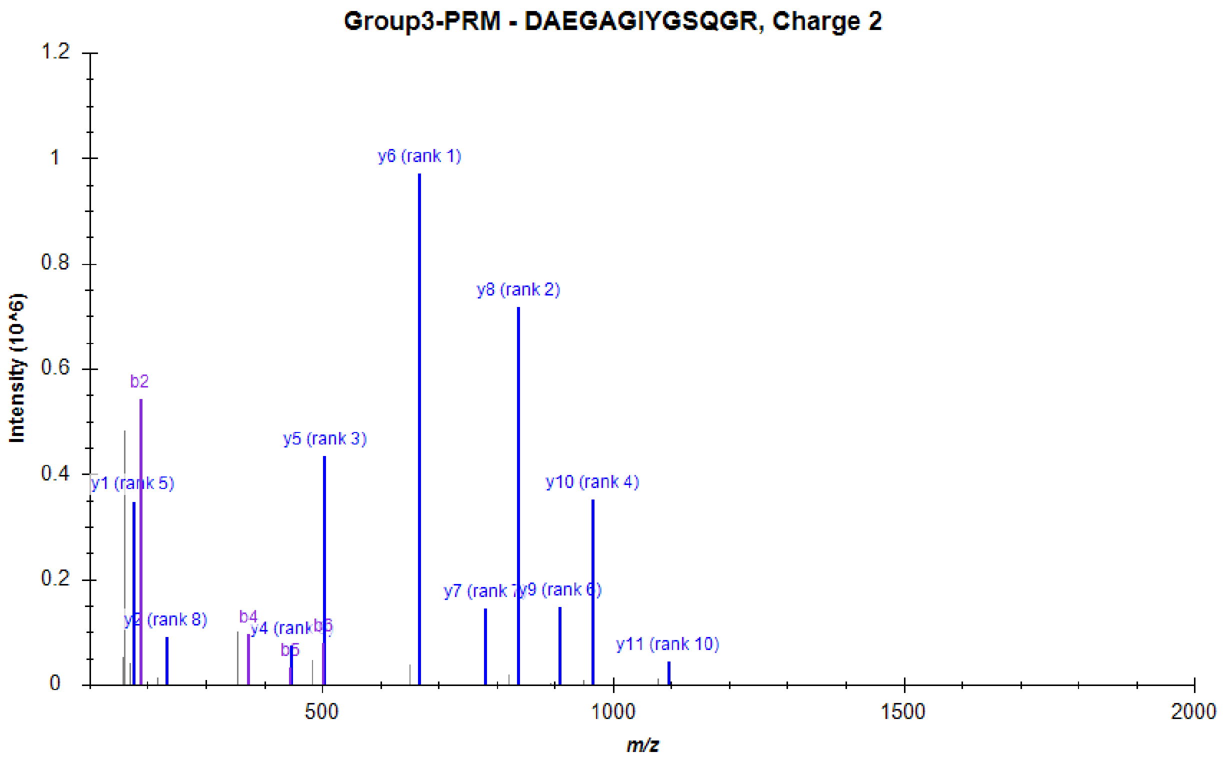
Group3-PRM - DAEGAGIYGSQGR, Charge 2
Intensity (10^6)
m/z
y6 (rank 1)
y8 (rank 2)
b2
y5 (rank 3)
y10 (rank 4)
(rank 8)
b4
b5
b6
y11 (rank 10)

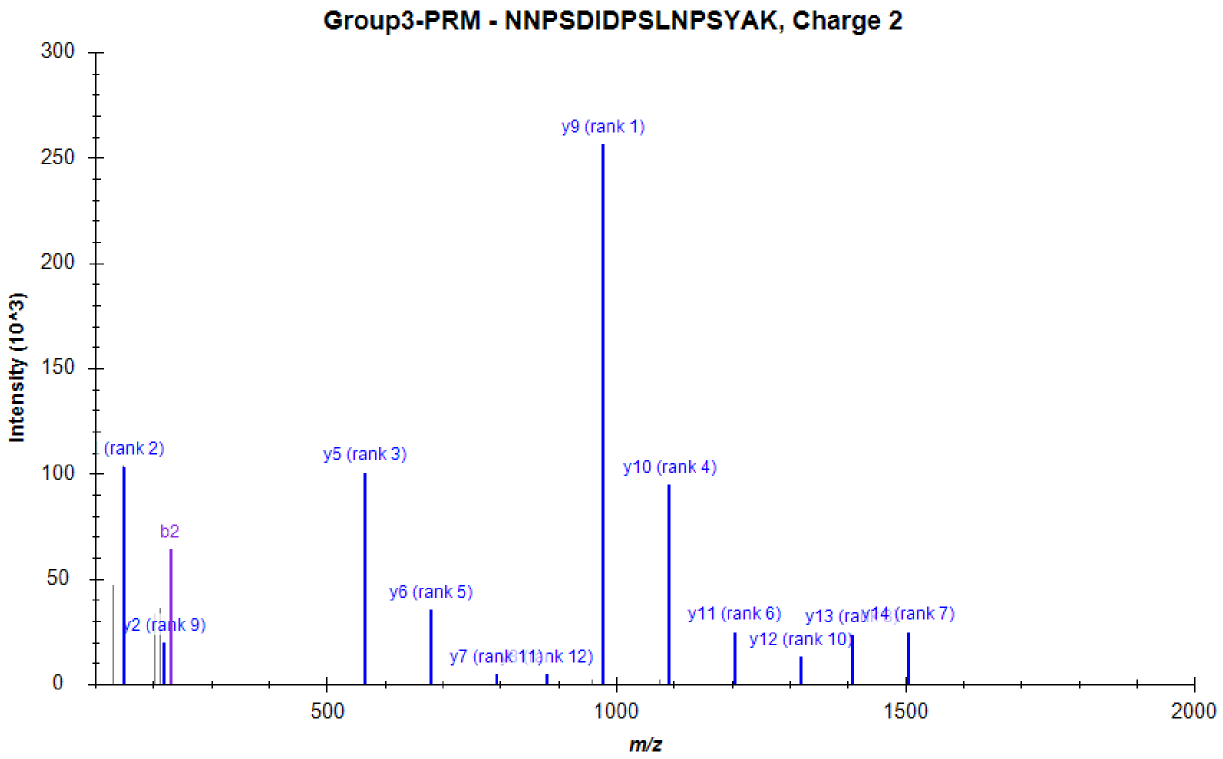
Group3-PRM - NNPSDIDPSLNPSYAK, Charge 2
Intensity (10^3)
m/z
y9 (rank 1)
(rank 2)
y5 (rank 3)
y10 (rank 4)
y6 (rank 5)
y11 (rank 6)
y12 (rank 10)
y2 (rank 9)
b2
0
50
100
150
200
250
300
500
1000
1500
2000

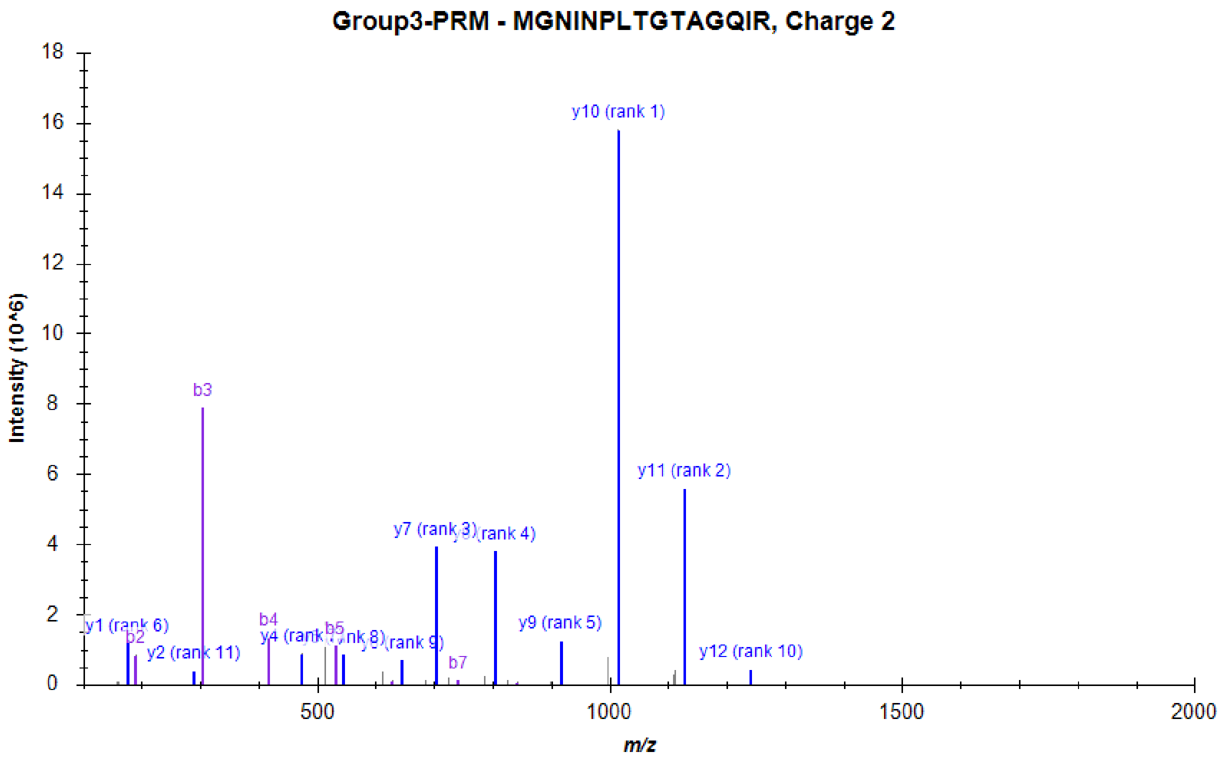
Group3-PRM - MGNINPLTGTAGQIR, Charge 2
Intensity (10^6)
m/z
y10 (rank 1)
y11 (rank 2)
y7 (rank 3)
y9 (rank 5)
y1 (rank 6)
y2 (rank 11)
y12 (rank 10)
b3
b4
b5
b7
b2
0
2
4
6
8
10
12
14
16
18
500
1000
1500
2000

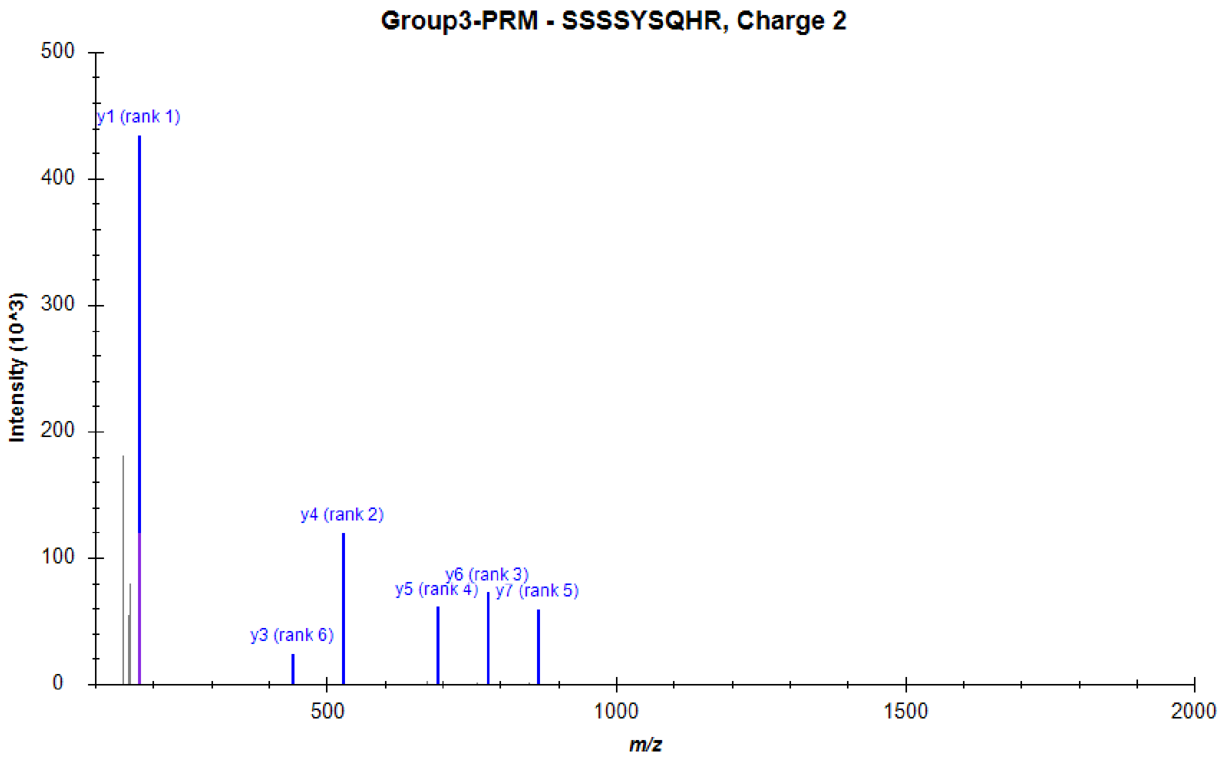
Group3-PRM - SSSSYSQHR, Charge 2
Intensity (10^3)
y1 (rank 1)
y4 (rank 2)
y6 (rank 3)
y5 (rank 4)
y7 (rank 5)
y3 (rank 6)
m/z
0
100
200
300
400
500
1000
1500
2000

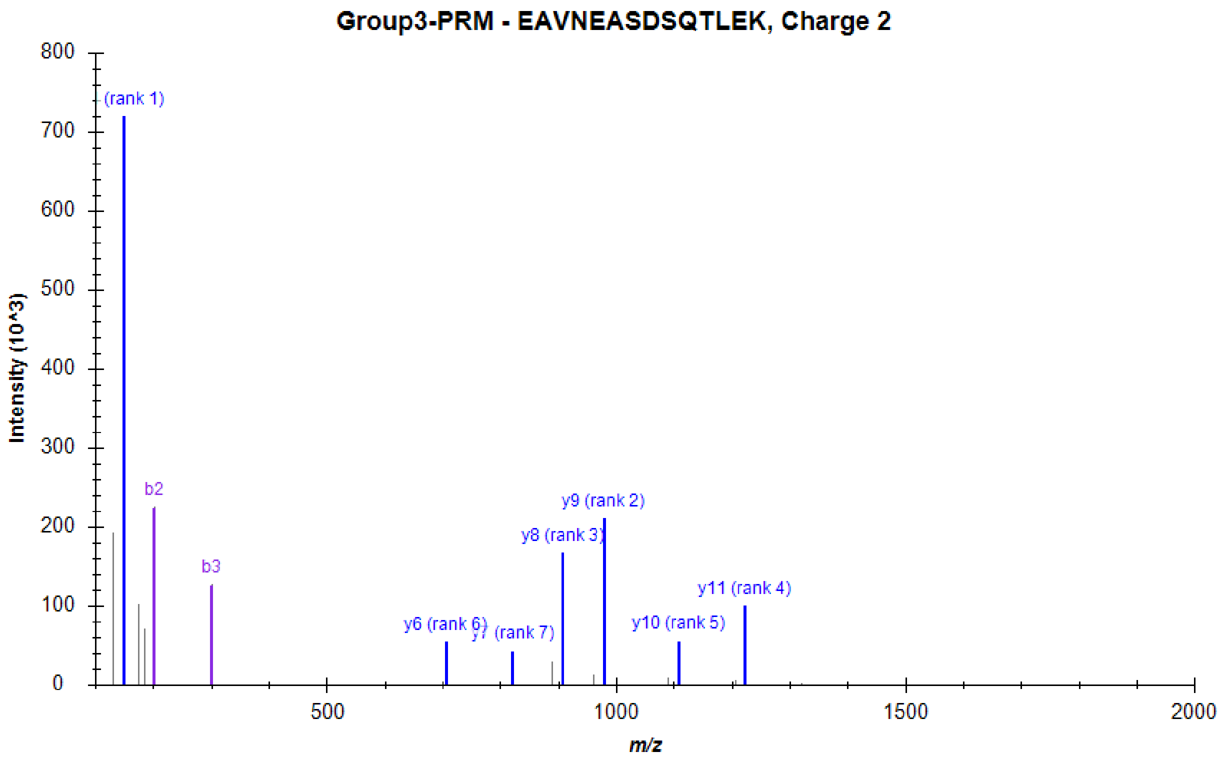
Group3-PRM - EAVNEASDSQTLEK, Charge 2
Intensity (10^3)
(rank 1)
b2
b3
y9 (rank 2)
y8 (rank 3)
y11 (rank 4)
y10 (rank 5)
y6 (rank 6)
y7 (rank 7)
m/z
0
100
200
300
400
500
600
700
800
1000
1500
2000

Group3-PRM - ELCSETGADQEDALAR, Charge 2

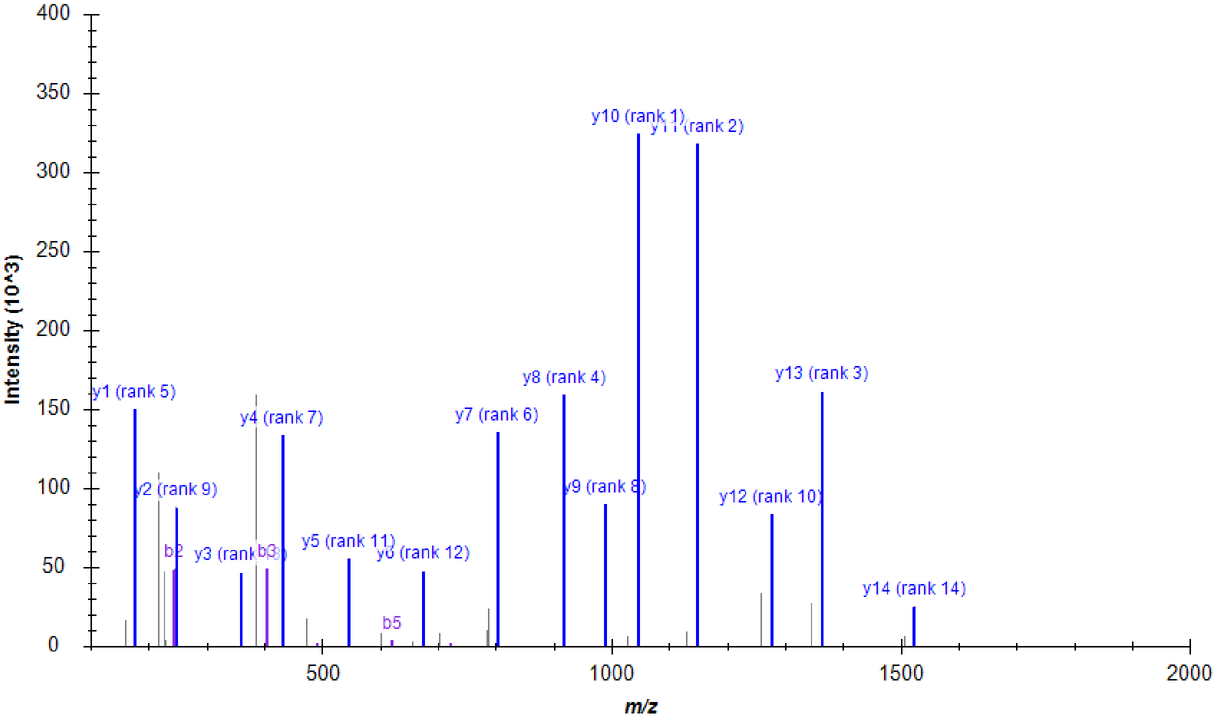

Group3-PRM - DIEEELGAPR, Charge 2

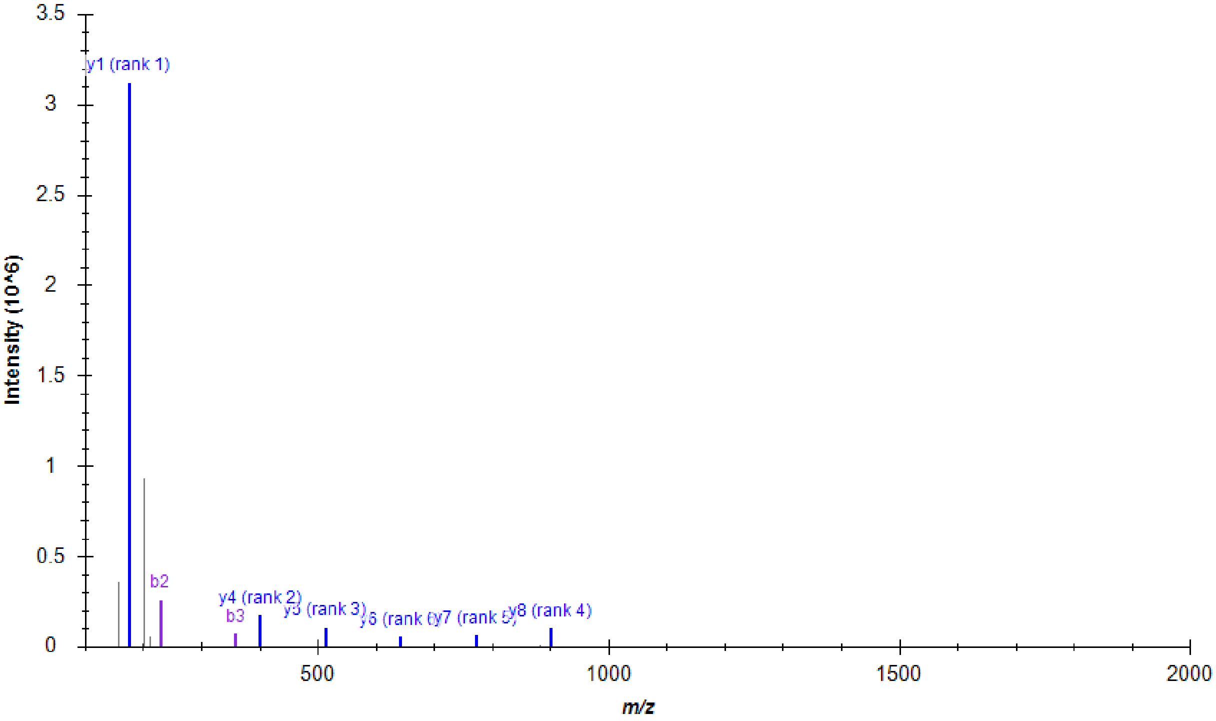

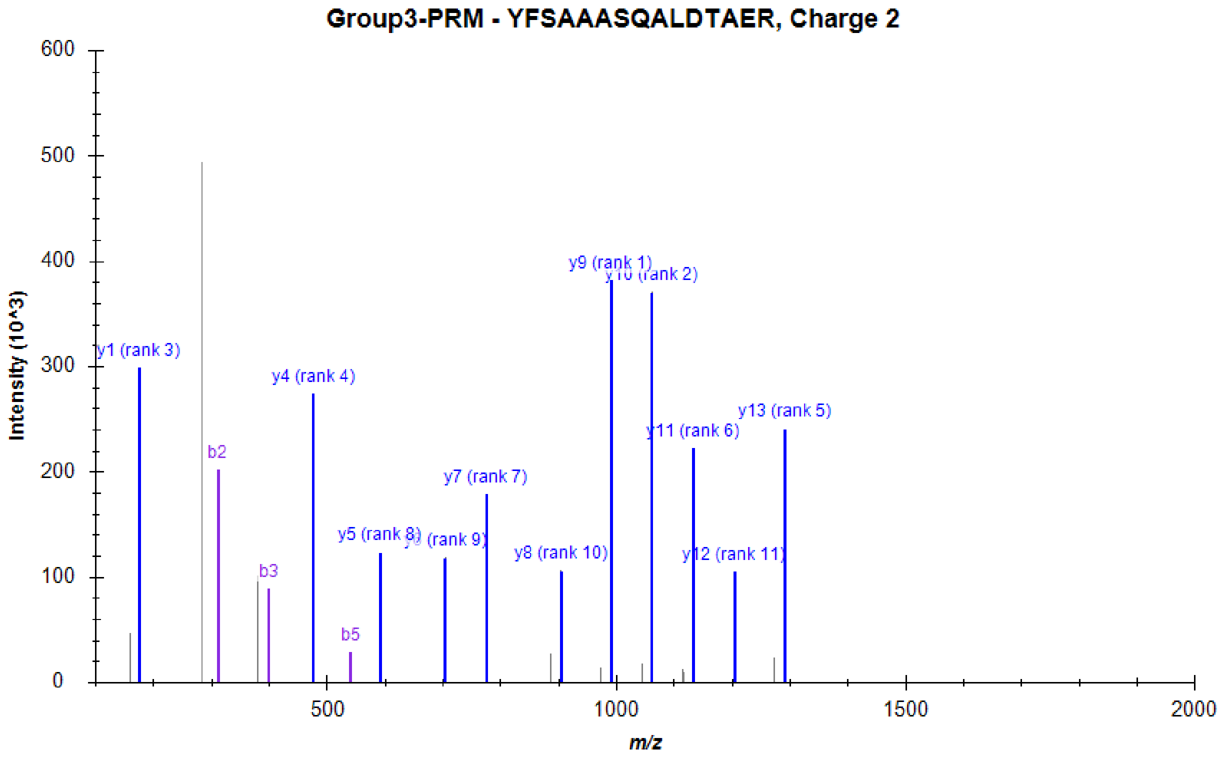
Group3-PRM - YFSAAASQALDTAER, Charge 2
Intensity (10^3)
m/z
600
500
400
300
200
100
0
500
1000
1500
2000
y1 (rank 3)
b2
b3
y4 (rank 4)
b5
y5 (rank 8)
(rank 9)
y7 (rank 7)
y8 (rank 10)
y9 (rank 1)
(rank 2)
y11 (rank 6)
y12 (rank 11)
y13 (rank 5)
